# The associations between gut microbiota and chronic respiratory diseases: a Mendelian randomization study

**DOI:** 10.3389/fmicb.2023.1200937

**Published:** 2023-06-02

**Authors:** Hanyu Shi, Tong Zhao, RuiHui Geng, Liang Sun, Haojun Fan

**Affiliations:** ^1^Department of Internal Medicine, Hospital of the First Mobile Corps of the Chinese People’s Armed Police Force, Dingzhou, Hebei, China; ^2^Department of Pulmonary and Critical Care, Characteristic Medical Center of the Chinese People’s Armed Police Force, Tianjin, China; ^3^Institute of Disaster and Emergency Medicine, Tianjin University, Tianjin, China

**Keywords:** gut microbiota, Mendelian randomization analaysis, chronic respiratory diseases, chronic obstructive pulmonary disease, asthma, idiopathic pulmonary fibrosis, sarcoidosis, pneumoconiosis

## Abstract

**Introduction:**

Growing evidence indicates that variations in the composition of the gut microbiota are linked to the onset and progression of chronic respiratory diseases (CRDs), albeit the causal relationship between the two remains unclear.

**Methods:**

We conducted a comprehensive two-sample Mendelian randomization (MR) analysis to investigate the relationship between gut microbiota and five main CRDs, including chronic obstructive pulmonary disease (COPD), asthma, idiopathic pulmonary fibrosis (IPF), sarcoidosis, and pneumoconiosis. For MR analysis, the inverse variance weighted (IVW) method was utilized as the primary method. The MR–Egger, weighted median, and MR-PRESSO statistical methods were used as a supplement. To detect heterogeneity and pleiotropy, the Cochrane and Rucker Q test, MR–Egger intercept test, and MR-PRESSO global test were then implemented. The leave-one-out strategy was also applied to assess the consistency of the MR results.

**Results:**

Based on substantial genetic data obtained from genome-wide association studies (GWAS) comprising 3,504,473 European participants, our study offers evidence that several gut microbial taxa, including 14 probable microbial taxa (specifically, 5, 3, 2, 3 and 1 for COPD, asthma, IPF, sarcoidosis, and pneumoconiosis, respectively) and 33 possible microbial taxa (specifically, 6, 7, 8, 7 and 5 for COPD, asthma, IPF, sarcoidosis, and pneumoconiosis, respectively) play significant roles in the formation of CRDs.

**Discussion:**

This work implies causal relationships between the gut microbiota and CRDs, thereby shedding new light on the gut microbiota-mediated prevention of CRDs.

## 1. Brief summary

### 1.1. Evidence before this study

Alterations in the formation of gut microbiota are closely linked to chronic respiratory diseases (CRDs). It is imperative to determine whether gut microbes have a causal relationship with the development of CRDs or if they are simply a result of shared risk factors.

### 1.2. Added value of this study

The study utilized two-sample Mendelian randomization (MR) analysis, a novel statistical method, to investigate the correlation between the gut microbiota and five prevalent CRDs, including chronic obstructive pulmonary disease (COPD), asthma, idiopathic pulmonary fibrosis (IPF), sarcoidosis, and pneumoconiosis. Our study, which analyzed genetic data from 3,504,473 European participants through genome-wide association studies (GWAS), provides evidence that numerous gut microbial taxa, including 14 probable and 33 possible microbial taxa, play important roles in the formation of CRDs.

### 1.3. Implications of all the available evidence

This work implies causal relationships between the gut microbiota and CRDs, thereby shedding new light on the gut microbiota-mediated prevention of CRDs.

## 2. Introduction

Chronic respiratory diseases(CRDs), which affect the airways and other lung structures, are among the leading causes of morbidity and mortality worldwide. Chronic obstructive pulmonary disease (COPD), asthma, interstitial lung disease (ILD), sarcoidosis and occupational lung diseases are among the most prevalent chronic respiratory conditions. These diseases are huge contributors to the escalating global burden of noncommunicable diseases (NCDs; Collaborators GBDCRD, 2020) and have grown into a major threat to public health in all nations, especially those with developing economies and low-income regions (Collaborators GBDCRD, 2020; Hussain et al., 2021). Current data indicate that the number of individuals worldwide afflicted by chronic respiratory illnesses has surged by 39.8% since 1990, reaching nearly 545 million in 2017 (Labaki and Han, 2020). Notably, chronic respiratory illnesses caused 3.8 million fatalities in 2016, representing 9% of all NCD fatalities and 7% of all deaths globally (Collaborators GBDCRD, 2020).

Although the pathogenesis and etiology of CRDs are not fully understood, genetic and environmental factors are of major importance in their development. In addition, accumulating evidence suggests that alterations in the formation of gut microbiota are closely associated with CRDs (Chunxi et al., 2020). The human gut microbiota is a complex, dynamic, and spatially heterogeneous ecosystem inhabited by a myriad of microorganisms, including bacteria and fungi, that interact with each other and with the human host (Gomaa, 2020). Gut microbiota dysbiosis not only modulates the immune responses of the gastrointestinal (GI) tract but also impacts the immunity of distal organs, such as the lung, further affecting lung health and respiratory diseases, which led to the coining of the gut-lung axis concept (Zhou et al., 2021). Recent studies have implicated gut microbial dysbiosis in the etiology and pathogenesis of common respiratory disorders such as asthma, COPD, and IPF (Li et al., 2021; Saint-Criq et al., 2021; Shi et al., 2021). However, our understanding of the mechanism involving the gut-lung axis is still in its infancy and requires more clarification (Chunxi et al., 2020; Zhou et al., 2021). It is essential to determine whether gut microbes play causal roles in the development of CRDs or merely serve as consequences of a shared risk factor profile.

Mendelian randomization (MR) is a recently developed statistical method for inferring causality that mimics a randomized controlled trial because genetic variants are assigned randomly during conception (Birney, 2022). MR uses single nucleotide polymorphisms (SNPs) as instrumental variables to model and infer causal effects, thereby eliminating the influence of confounding variables. Moreover, since heredity is irreversible, it can eliminate the interference of reverse causation (Xu et al., 2021). MR has been widely applied to explore the association between gut microbiota and various diseases, including preeclampsia (Li et al., 2022), diabetic retinopathy (Liu et al., 2022), and psychiatric disorders (Ni et al., 2021), yet there is little evidence to investigate the causal linkages of gut microbiota on CRDs.

In this work, a comprehensive two-sample MR analysis was undertaken to determine the association between the gut microbiota and five common CRDs, including COPD, asthma, idiopathic pulmonary fibrosis (IPF), sarcoidosis, and pneumoconiosis. Our research sheds light on the potential role of the gut microbiota in the etiology of CRDs and may lead to the development of novel therapeutic options for these debilitating diseases.

## 3. Materials and methods

### 3.1. Study design

**Figure 1:**
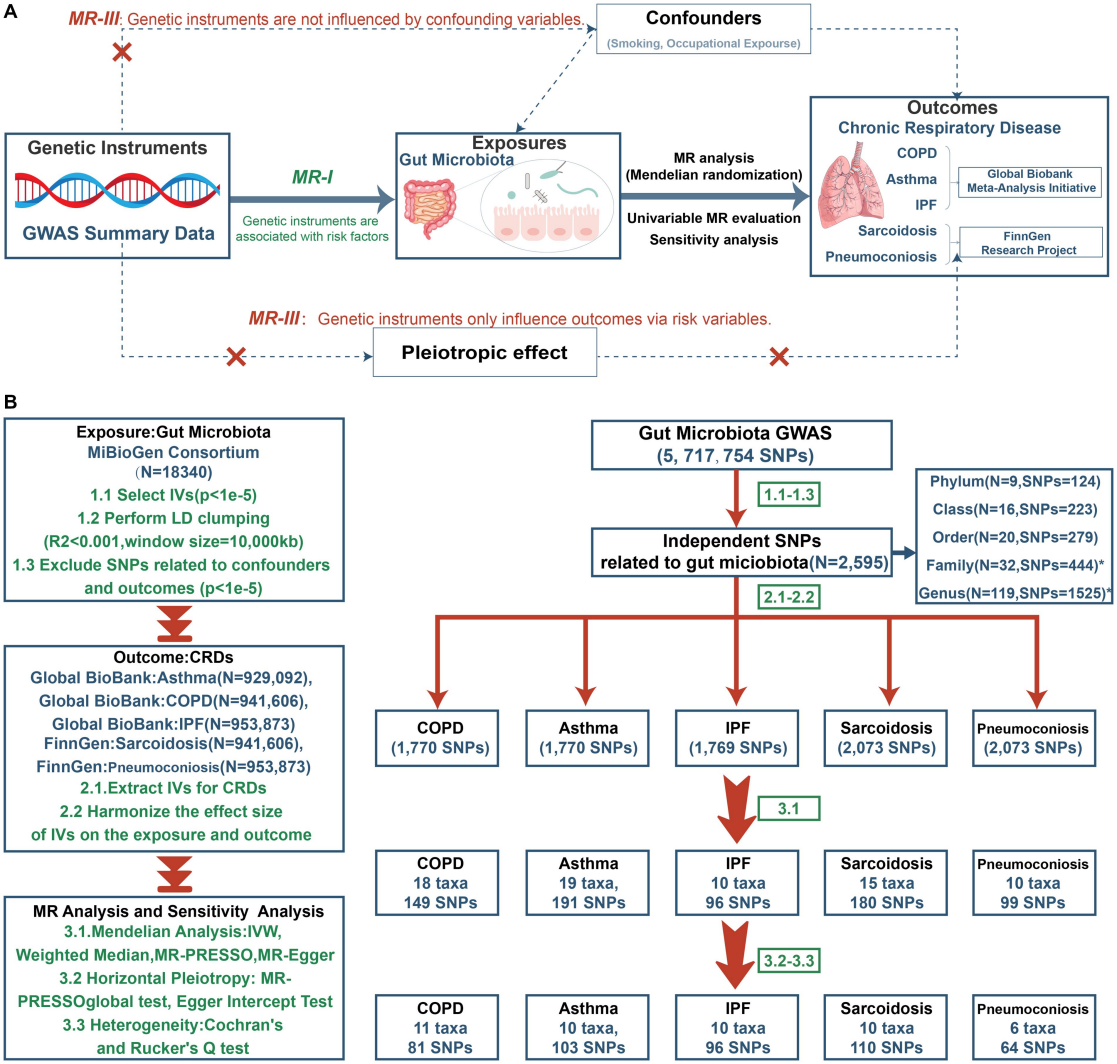
The study design of MR analysis **(A)** and the overall workflow **(B)**. GWAS, genome-wide association study; MR, mendelian randomization; IVs, instrument variables; LD, linkage disequilibrium; SNP, single nucleotide polymorphism; IVW, inverse-variance-weighted; MR-PRESSO, MR pleiotropy residual sum and outlier; COPD, chronic obstructive pulmonary disease; IPF, idiopathic pulmonary fibrosis.

A comprehensive two-sample Mendelian randomization was undertaken at five levels (including phylum, class, order, family and genus) to investigate the causative role of gut microbiota on five prevalent CRDs. Figure 1A presents the study design alongside the essential MR assumptions: (1) instrument variables (IVs) were associated with the exposure factors, (2) IVs were not related to any confounding factors, and (3) IVs only affected the outcome through the pathway of the exposure factors (Davies et al., 2018).

### 3.2. Data sources

The genetic information of gut microbiota as exposure was obtained from the largest genome-wide association study (GWAS) conducted by the MiBioGen consortium,^1^ which included 5,717,754 SNPs and 18,340 participants from 24 cohorts (total 211 taxa: 9 phylum, 16 classes, 20 orders, 35 families, and 131 genus; Kurilshikov et al., 2021). Furthermore, 15 taxa (12 genus and 3 families) with unknown groups were excluded, meaning that 196 bacterial taxa were included in the subsequent MR analysis.

GWAS summary statistics (Table 1) for the first three CRDs (COPD, asthma, IPF) were extracted from newly published GWAS meta-analyses from the Global Biobank Meta-Analysis Initiative (GBMI). The GWAS meta-analyses included 54,606 cases and 887,000 controls for COPD, 95554 cases and 833,538 controls for asthma, and 6,257 cases and 947,616 controls for IPF, which comprises nine biobanks (BioVU, Colorado Center for Personalized Medicine, Estonian Biobank, FinnGen, HUNT Study, Michigan Genomics Initiative, Mass General Brigham, UCLA Precision Health Biobank, and UK Biobank; Zhou et al., 2022). Additionally, the genetic data on sarcoidosis (3,597 cases and 337,121 controls) and pneumoconiosis (548 cases and 338,636 controls) were accessed from the eighth version of the FinnGen Biobank,^2^ a prospective cohort study involving 35,379,992 individuals (Kurki et al., 2023). Both databases were adopted due to their largest sample size of GWAS data currently available for these conditions.

**Table 1:** Characteristics of the GWAS used for analyses.

Trait	Data Type	N_cases	N_controls	Ethnicity	Consortium	PubMed ID
COPD	Outcome	54,606	887,000	European	GBMI	36,777,996
Asthma	Outcome	95,554	833,538	European	GBMI	36,777,996
IPF	Outcome	6,257	947,616	European	GBMI	36,777,996
Sarcoidosis	Outcome	3,597	337,121	European	FinnGen_r8	36,653,562
Pneumoconiosis	Outcome	548	338,636	European	FinnGen_r8	36,653,562
Gut Microbiota	Exposure	18,340		European	MiBioGen	33,462,485

There were few overlapping samples or closely related individuals between the gut microbiota and CRDs (Supplementary Table S1). At the database level, there were no significant overlaps between the samples. We then calculated the sample overlap at the country level and found the maximum overlap rate to be just 0.0102, further guaranteeing the independence of samples between exposure and outcome. The original GWAS were approved by their respective institutions, and all the data used in our study were publicly available; no additional ethical approval was needed.

### 3.3. Instrument variables selection

To ensure the accuracy and reliability of the causal relationship between the gut microbiota and CRDs, we conducted a series of stringent quality tests to pick IVs that met the three assumptions of MR analysis. (1) Given the limited number of available SNPs, we selected SNPs significantly related to the gut microbiota with a loose cutoff of *p* < 1e-5 (Yu et al., 2023). Then, we clumped genetic variations within 10,000 kb at the level of linkage disequilibrium (LD) *r*^2^ = 0.001. The F statistic (beta^2^/se^2^) was calculated to measure the statistical strength of each SNP, and those with an *F* value <10 were removed for weak strength (MR hypothesis I) (Xie et al., 2023). (2) The SNPs that were significantly associated with the outcomes (*p* < 1e-5) were eliminated (MR hypothesis III). (3) We searched all eligible SNPs using PhenoScanner^3^ to exclude SNPs relevant to potential confounders such as smoking and occupational exposure (MR hypothesis II; Kamat et al., 2019).

### 3.4. MR analysis

We conducted an MR study to investigate the causal link between the gut microbiota and five prevalent CRDs (COPD, asthma, IPF, sarcoidosis, and pneumoconiosis). Four popular MR methods were employed, including the random-effect inverse-variance-weighted (IVW) test, the weighted median (WM), Mendelian randomization pleiotropy residual sum and outlier (MR-PRESSO), and the MR-Egger regression.

On the assumption that each genetic variant satisfies the IV assumptions, the IVW method was employed to incorporate the Wald ratio assessments of each instrumental variable into a meta-analysis, which is equivalent to conducting a weighted linear regression of the associations between the instrumental variables. The IVW method was reported to be advantageous since it offers estimates that are not influenced by horizontal pleiotropy (Burgess et al., 2013). Second, assuming at least 50% of the selected SNPs are legitimate, the weighted median estimator can yield unbiased causal effects (Bowden et al., 2016). Third, the MR–Egger sensitivity estimator can generate unbiased estimates of causality relationships even if all instrumental SNPs are invalid due to pleiotropy (Bowden et al., 2015). Fourth, the MR-PRESSO method was implemented because it can discover pleiotropic outliers, and after eliminating outliers, the causal impact estimate is obtained using the inverse-variance–weighted method (Verbanck et al., 2018). If the outcomes of these approaches are incongruent, we will prioritize IVW as the primary result. To ensure that each IV was correlated with the same effect allele, we harmonized the summary statistics and eliminated palindromic SNPs.

Moreover, we conducted a series of sensitivity analyzes to guarantee the authenticity and robustness of the results. On the one hand, the MR-PRESSO global test and the MR Egger intercept test were employed to evaluate the IVs’ global horizontal pleiotropy. *p* values greater than 0.05 for both methods revealed no horizontal pleiotropy (Verbanck et al., 2018). On the other hand, Cochran’s Q statistic (MR-IVW) and Rucker’s Q statistic (MR Egger) were utilized to identify heterogeneity in this MR analysis, and *p* > 0.05 indicated that there was no heterogeneity (Hemani et al., 2018). Finally, a leave-one-out sensitivity test was used to identify whether a single SNP influenced the inference of causal associations.

### 3.5. Statistical analysis

To obtain a more stringent interpretation of the causal link, we additionally applied the Bonferroni-corrected significance criterion, defined as *p* = 0.05/n, at each feature level (phylum: 0.05/9, class: 0.05/16, order: 0.05/20, family: 0.05/32, and genus: 0.05/119). Microbiomes with *p* values less than 0.05/n were deemed to have a highly probable relationship with CRDs, while those that displayed nominal significance (0.05) after three main MR analyzes (IVW, WM, MR-PRESSO) but lost significance after adjustment were regarded as probable features. Microbiomes with *p* values <0.05 in less than three MR analyzes were considered to have possible relationships (Yu et al., 2021; Long et al., 2023; Xie et al., 2023). The statistical analyses were performed using R version 4.1.3 (R Foundation for Statistical Computing). “TwoSampleMR,” “MRInstruments,” and “MendelianRandomization” are the most frequently employed R packages.

## 4. Results

### 4.1. Overview

**Figure 2:**
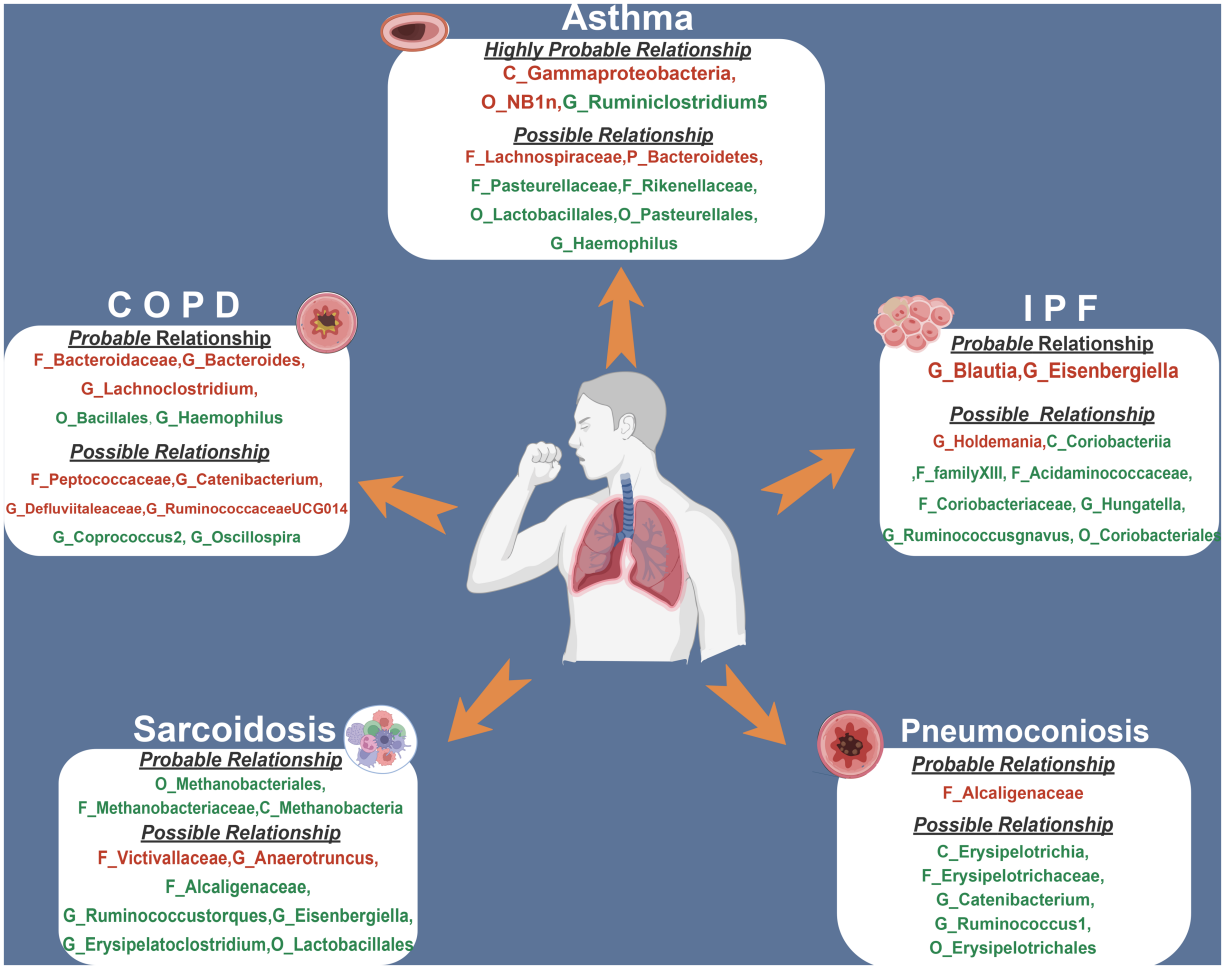
The summary of MR results of significant relationship between gut microbiota and CRDs. The red indicates risk factors, while the green illustrates protection. COPD, chronic obstructive pulmonary disease; IPF, idiopathic pulmonary fibrosis.

Figure 1B depicts the study’s overall workflow. After screening for SNPs linked with exposure and removing LD, we obtained 2,601 SNPs of 196 taxa and then removed 4 SNPs associated with outcomes (rs11597285, rs62240188, rs62028349, rs12925026) and 2 SNPs connected with the confounding factor smoking (rs4506202, rs12288512). Finally, 2,595 SNPs from 196 taxa were employed as IVs, and the F statistics for each SNP ranged from 16.91 to 36.57, indicating that no instrument bias was present. 717 SNPs from 72 taxa were obtained after harmonizing exposure and outcome alleles and performing MR analysis (Supplementary Table S2). We identified 454 SNPs across 47 taxa after conducting numerous sensitivity analyzes (Supplementary Table S3). Figure 2 summarizes the conclusive findings between gut microbiota and CRDs.

### 4.2. Causal relationship between gut microbiota and COPD

**Figure 3:**
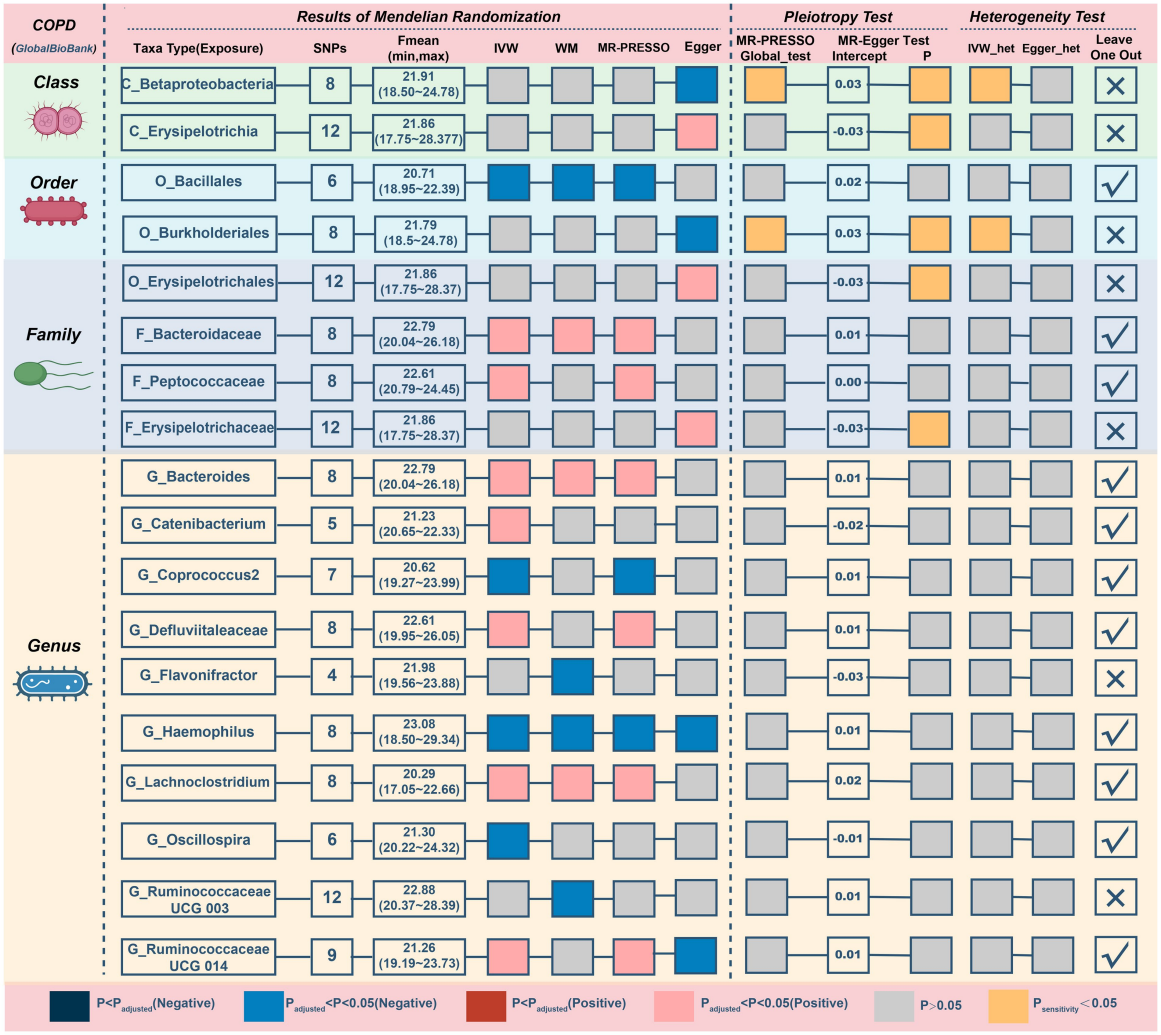
MR results and sensitivity analysis of significant relationship between gut microbiota and COPD. COPD, chronic obstructive pulmonary disease; MR, Mendelian randomization; SNP, single nucleotide polymorphism; IVW, inverse-variance-weighted; MR-PRESSO, MR pleiotropy residual sum and outlier.

This study discovered 5 probable traits in the development of COPD, one of which belonged to orders, two to families, and two to genus (Figure 3; Table 2). Higher genetically predicted levels of the *family Bacteroidaceae* (IVM: OR = 1.118, 95% CI 1.016–1.229, *p* = 0.022; WM: OR = 1.174, 95% CI 1.038–1.328, *p* = 0.011; MR-PRESSO: OR = 1.118, 95% CI 1.019–1.225, *p* = 0.049), *genus Bacteroides*(IVM: OR = 1.118, 95% CI 1.016–1.229, p = 0.022; WM: OR = 1.174, 95% CI 1.033–1.333, *p* = 0.014; MR-PRESSO: OR = 1.118, 95% CI 1.019–1.225, p = 0.049), and *genus Lachnoclostridium* (IVM: OR = 1.173, 95% CI 1.045–1.316, *p* = 0.007; WM: OR = 1.165, 95% CI 1.017–1.334, *p* = 0.027; MR-PRESSO: OR = 1.173, 95% CI 1.045–1.316, *p* = 0.030) were significantly linked with an elevated risk of COPD. In contrast, elevated genetically predicted levels of the *order Bacillales* (IVW: OR = 0.938, 95% CI 0.895–0.984, *p* = 0.008; WM: OR = 0.925, 95% CI 0.871–0.983, *p* = 0.011; MR-PRESSO: OR = 0.938, 95% CI 0.901–0.977, *p* = 0.028)and the *genus Haemophilus* (IVM: OR = 0.925, 95% CI 0.874–0.98, p = 0.008; WM: OR = 0.906, 95% CI 0.837–0.98, *p* = 0.014; MR-PRESSO: OR = 0.925, 95% CI 0.874–0.98, *p* = 0.033) were substantially discharged to a lower risk level. Additionally, a possible relationship between the 13 taxa and COPD was observed.

**Table 2:** MR results of significant relationship between gut microbiota and COPD.

Exposures on COPD	SNPs	IVW		WM		MR-Presso		MR-Egger	
		OR (95% CI)	*p*	OR (95% CI)	*p*	OR (95% CI)	*p*	OR (95% CI)	*p*
C_Betaproteobacteria	8	0.981 (0.849–1.134)	0.797	0.917 (0.796–1.057)	0.231	0.981 (0.849–1.134)	0.805	0.626 (0.459–0.855)	**0.026**
C_Erysipelotrichia	12	0.997 (0.912–1.091)	0.950	0.999 (0.894–1.116)	0.983	0.997 (0.912–1.091)	0.951	1.552 (1.101–2.187)	**0.031**
F_Bacteroidaceae	8	1.118 (1.016–1.229)	**0.022**	1.174 (1.038–1.328)	**0.011**	1.118 (1.019–1.225)	**0.049**	0.922 (0.528–1.608)	0.784
F_Erysipelotrichaceae	12	0.997 (0.912–1.091)	0.950	0.999 (0.889–1.123)	0.983	0.997 (0.912–1.091)	0.951	1.552 (1.101–2.187)	**0.031**
F_Peptococcaceae	8	1.08 (1.013–1.151)	**0.018**	1.056 (0.97–1.149)	0.212	1.08 (1.042–1.118)	**0.004**	1.097 (0.946–1.273)	0.266
G_Bacteroides	8	1.118 (1.016–1.229)	**0.022**	1.174 (1.033–1.333)	**0.014**	1.118 (1.019–1.225)	**0.049**	0.922 (0.528–1.608)	0.784
G_Catenibacterium	5	1.057 (1.002–1.114)	**0.042**	1.036 (0.966–1.11)	0.324	1.057 (1.016–1.099)	0.052	1.19 (0.727–1.949)	0.538
G_Coprococcus2	7	0.919 (0.846–1)	**0.049**	0.919 (0.825–1.025)	0.130	0.919 (0.88–0.961)	**0.010**	0.805 (0.419–1.546)	0.543
G_Defluviitaleaceae	8	1.076 (1.01–1.145)	**0.023**	1.058 (0.978–1.145)	0.161	1.076 (1.034–1.119)	**0.008**	1.004 (0.81–1.243)	0.975
G_Flavonifractor	4	0.887 (0.769–1.023)	0.098	0.865 (0.754–0.992)	**0.037**	0.887 (0.769–1.023)	0.197	1.176 (0.697–1.983)	0.605
G_Haemophilus	8	0.925 (0.874–0.98)	**0.008**	0.906 (0.837–0.98)	**0.014**	0.925 (0.874–0.98)	**0.033**	0.838 (0.742–0.947)	**0.030**
G_Lachnoclostridium	8	1.173 (1.045–1.316)	**0.007**	1.165 (1.017–1.334)	**0.027**	1.173 (1.045–1.316)	**0.030**	0.921 (0.635–1.336)	0.681
G_Oscillospira	6	0.91 (0.837–0.99)	**0.029**	0.913 (0.815–1.022)	0.115	0.91 (0.837–0.99)	0.079	1.045 (0.726–1.505)	0.824
G_RuminococcaceaeUCG003	12	0.993 (0.908–1.086)	0.880	0.895 (0.803–0.997)	**0.043**	0.993 (0.908–1.086)	0.882	0.844 (0.639–1.116)	0.262
G_RuminococcaceaeUCG014	9	1.091 (1.013–1.174)	**0.021**	1.056 (0.953–1.171)	0.300	1.091 (1.026–1.159)	**0.024**	1.022 (0.853–1.225)	0.821
O_Bacillales	6	0.938 (0.895–0.984)	**0.008**	0.925 (0.871–0.983)	**0.011**	0.938 (0.901–0.977)	**0.028**	0.84 (0.688–1.026)	0.163
O_Burkholderiales	8	0.981 (0.85–1.132)	0.793	0.917 (0.797–1.055)	0.224	0.981 (0.85–1.132)	0.800	0.634 (0.464–0.865)	**0.028**
O_Erysipelotrichales	12	0.997 (0.912–1.091)	0.950	0.999 (0.891–1.12)	0.983	0.997 (0.912–1.091)	0.951	1.552 (1.101–2.187)	**0.031**

All data with *p <* 0.05 are in bold. COPD, chronic obstructive pulmonary disease; MR, Mendelian randomization; SNP, single nucleotide polymorphism; CI, confidence interval; IVW, inverse-variance-weighted; WM, weighted median; MR-PRESSO, MR pleiotropy residual sum and outlier; OR, odd ratio; P_: phylum; C_: class; O_: order; F_: family; G_: genus.

The MR–Egger intercept (Figure 3; Supplementary Figure S1) and MR-PRESSO global tests revealed that five possible taxa exhibited horizontal pleiotropy (*class Betaproteobacteria, class Erysipelotrichia, family Erysipelotrichaceae, order Burkholderiales, and order Erysipelotrichales, p* < 0.05). Cochrane’s Q test and Rucker’s Q statistic revealed that there was no discernible heterogeneity among the selected SNPs in the remaining taxa (*p* > 0.05; Figure 3). Nonetheless, the leave-one-out analysis (Supplementary Figure S2) revealed that a few particular SNPs may have overlooked the positive results of two other possible taxa (*genus Flavonifractor, genus RuminococcaceaeUCG003*). Following the removal of 7 unsteady features, our analysis identified 5 probable (3 hazardous and 2 protective features) and 6 possible (4 hazardous and 2 protective features) taxa on COPD.

### 4.3. Causal relationship between gut microbiota and asthma

**Figure 4:**
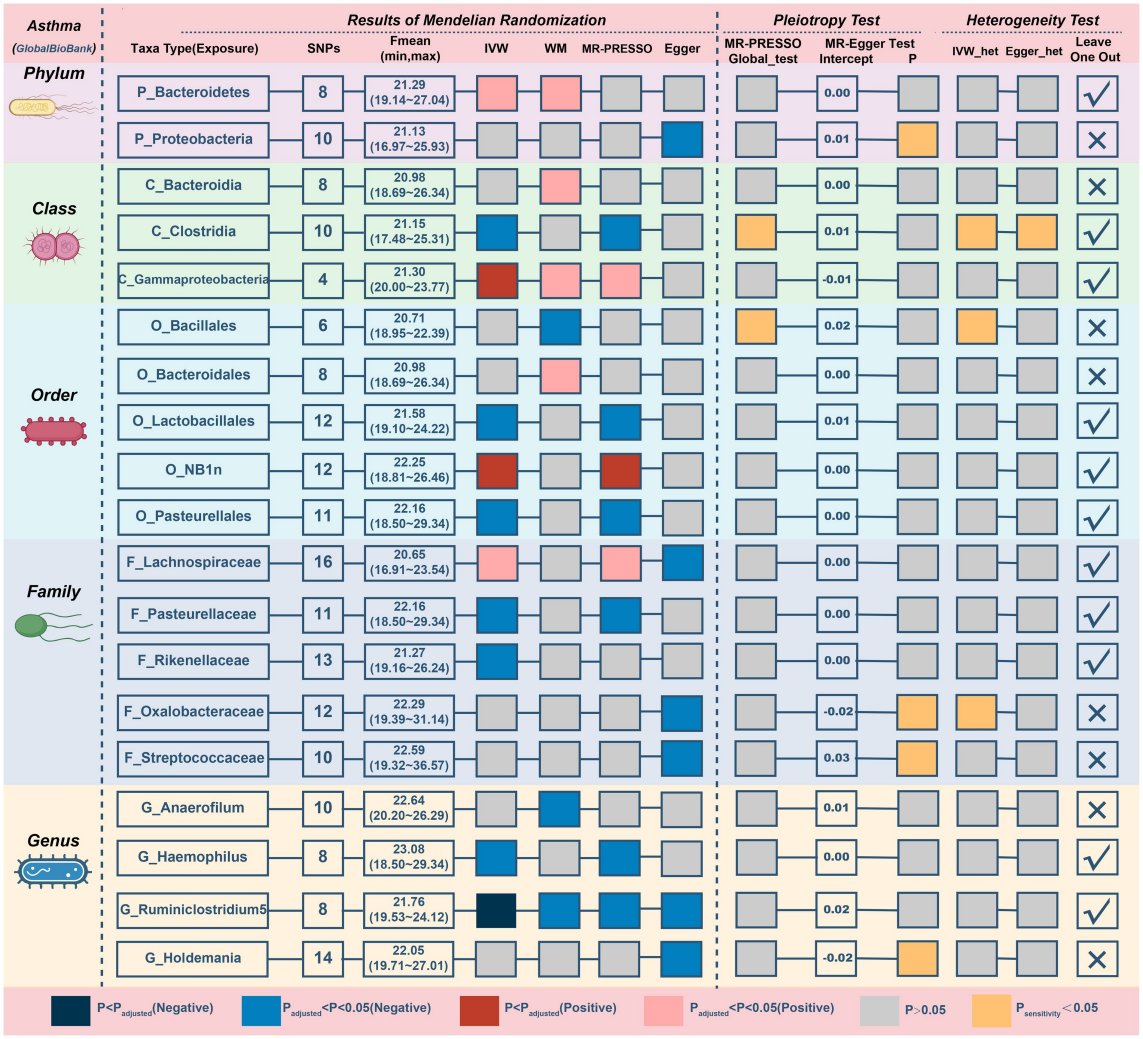
MR results and sensitivity analysis of significant relationship between gut microbiota and asthma. MR, Mendelian randomization; SNP, single nucleotide polymorphism; IVW, inverse-variance-weighted; MR-PRESSO, MR pleiotropy residual sum and outlier.

Results from the Bonferroni-corrected test (Figure 4; Table 3) identified higher levels of *class Gammaproteobacteria* (IVM: OR = 1.15, 95% CI 1.049–1.26, *p* = 0.003; WM: OR = 1.143, 95% CI 1.024–1.276, *p* = 0.018; MR-PRESSO: OR = 1.15, 95% CI 1.112–1.189, *p* = 0.004) and *order NB1n* (IVM: OR = 1.064, 95% CI 1.032–1.096, *p* = 5.82E-05; WM: OR = 1.043, 95% CI 1–1.089, *p* = 0.052; MR-PRESSO: OR = 1.064, 95% CI 1.032–1.096, *p* = 0.002) suggests a highly probable relationship with higher risk of asthma, whereas a higher level of *genus Ruminiclostridium5* (IVM: OR = 0.868, 95% CI 0.811–0.931, *p* = 6.24E-05; WM: OR = 0.89, 95% CI 0.81–0.978, *p* = 0.015; MR-PRESSO: OR = 0.868, 95% CI 0.811–0.931, *p* = 0.005) retains a highly probable protective relationship with asthma. In addition, there was a possible association between the 16 taxa and asthma.

**Table 3:** MR results of significant relationship between gut microbiota and asthma.

Exposures on Asthma	SNPs	IVW		WM		MR-Presso		MR-Egger	
		OR (95% CI)	*p*	OR (95% CI)	*p*	OR (95% CI)	*p*	OR (95% CI)	*p*
C_Bacteroidia	8	1.077 (0.989–1.172)	0.088	1.122 (1.012–1.243)	**0.028**	1.077 (0.989–1.172)	0.132	1.094 (0.837–1.43)	0.534
C_Clostridia	10	0.898 (0.819–0.986)	**0.023**	0.97 (0.889–1.058)	0.489	0.898 (0.819–0.986)	**0.049**	0.739 (0.471–1.161)	0.226
C_Gammaproteobacteria	4	1.15 (1.049–1.26)	**0.003**	1.143 (1.024–1.276)	**0.018**	1.15 (1.112–1.189)	**0.004**	1.4 (0.749–2.62)	0.402
F_Lachnospiraceae	16	1.079 (1.018–1.143)	**0.010**	1.061 (0.989–1.139)	0.097	1.079 (1.018–1.143)	**0.021**	1.103 (0.944–1.288)	0.238
F_Oxalobacteraceae	12	0.976 (0.939–1.014)	0.218	0.993 (0.949–1.039)	0.762	0.976 (0.939–1.014)	0.243	1.171 (1.039–1.32)	**0.027**
F_Pasteurellaceae	11	0.958 (0.925–0.992)	**0.015**	0.957 (0.912–1.004)	0.070	0.958 (0.934–0.982)	**0.007**	0.993 (0.922–1.069)	0.855
F_Rikenellaceae	13	0.936 (0.878–0.998)	**0.043**	0.951 (0.879–1.03)	0.218	0.936 (0.878–0.998)	0.066	0.993 (0.806–1.224)	0.951
F_Streptococcaceae	10	0.984 (0.912–1.062)	0.679	0.973 (0.898–1.055)	0.511	0.984 (0.912–1.062)	0.688	0.68 (0.539–0.859)	**0.012**
G_Anaerofilum	10	0.967 (0.927–1.01)	0.131	0.947 (0.902–0.994)	**0.029**	0.967 (0.927–1.01)	0.165	0.856 (0.686–1.069)	0.208
G_Haemophilus	8	0.958 (0.921–0.996)	**0.032**	0.967 (0.918–1.018)	0.200	0.958 (0.937–0.98)	**0.007**	0.956 (0.877–1.042)	0.347
G_Holdemania	14	0.985 (0.942–1.029)	0.492	0.999 (0.95–1.051)	0.982	0.985 (0.942–1.029)	0.504	1.134 (1.022–1.259)	**0.036**
G_Ruminiclostridium5	8	0.868 (0.811–0.931)	**6.241E-5**	0.89 (0.81–0.978)	**0.015**	0.868 (0.811–0.931)	**0.005**	0.693 (0.536–0.897)	**0.032**
O_Bacillales	6	0.977 (0.923–1.034)	0.417	0.937 (0.896–0.98)	**0.004**	0.977 (0.923–1.034)	0.454	0.822 (0.673–1.004)	0.127
O_Bacteroidales	8	1.077 (0.989–1.172)	0.088	1.122 (1.021–1.233)	**0.017**	1.077 (0.989–1.172)	0.132	1.094 (0.837–1.43)	0.534
O_Lactobacillales	12	0.93 (0.885–0.977)	**0.004**	0.945 (0.878–1.018)	0.135	0.93 (0.885–0.977)	**0.014**	0.848 (0.753–0.956)	**0.023**
O_NB1n	12	1.064 (1.032–1.096)	**5.821E-5**	1.043 (1–1.089)	0.052	1.064 (1.032–1.096)	**0.002**	1.056 (0.927–1.204)	0.431
O_Pasteurellales	11	0.958 (0.925–0.992)	**0.015**	0.957 (0.912–1.004)	0.070	0.958 (0.934–0.982)	**0.007**	0.993 (0.922–1.069)	0.855
P_Bacteroidetes	8	1.096 (1.011–1.187)	**0.025**	1.123 (1.022–1.234)	**0.016**	1.096 (1.011–1.187)	0.060	1.039 (0.81–1.331)	0.775
P_Proteobacteria	10	0.973 (0.909–1.041)	0.429	0.967 (0.892–1.048)	0.411	0.973 (0.909–1.041)	0.449	0.807 (0.685–0.951)	**0.034**

All data with *p* < 0.05 are in bold. MR, Mendelian randomization; SNP, single nucleotide polymorphism; CI, confidence interval; IVW, inverse-variance-weighted; WM, weighted median; MR-PRESSO, MR pleiotropy residual sum and outlier; OR, odd ratio; P_: phylum; C_: class; O_: order; F_: family; G_: genus.

The MR–Egger intercept (Supplementary Figure S3) and MR-PRESSO global tests demonstrated horizontal pleiotropy in six candidate taxa (phylum Proteobacteria, class Clostridia, order Bacillales, family Oxalobacteracea, family Streptococcaceae, and genus Holdemania). According to the Cochrane and Rucker Q tests, the remaining taxa showed negligible heterogeneity (Figure 4). In addition, the links of three possible taxa (class Bacteroidia, order Bacteroidales, genus Anaerofilum) were excluded because the leave-one-out analysis yielded inconsistent results (Supplementary Figure S4). In summary, our analysis identified 3 highly probable (2 harmful and 1 preventive features) and 7 possible (2 harmful and 5 preventive features) taxa associated with asthma.

### 4.4. Causal relationship between gut microbiota and IPF

**Figure 5:**
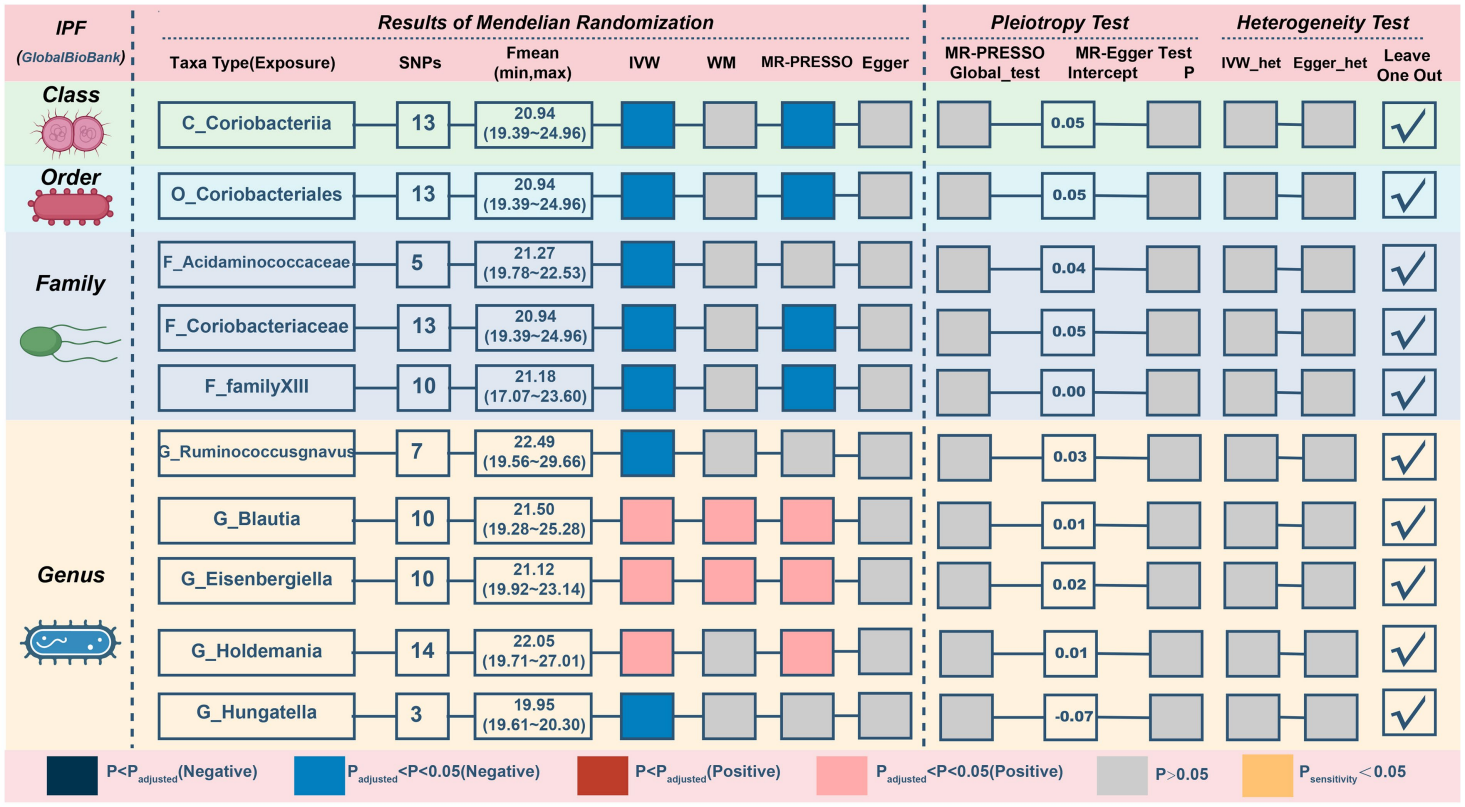
MR results and sensitivity analysis of significant relationship between gut microbiota and IPF. IPF, idiopathic pulmonary fibrosis; MR, Mendelian randomization; SNP, single nucleotide polymorphism; IVW, inverse-variance-weighted; MR-PRESSO, MR pleiotropy residual sum and outlier.

For IPF, only two microbiotas showed a probable association (Figure 5; Table 4). Increasing abundance of the *genus Blautia* (IVM: OR = 1.269, 95% CI 1.029–1.565, *p* = 0.026; WM: OR = 1.362, 95% CI 1.017–1.825, *p* = 0.038; MR-PRESSO: OR = 1.269, 95% CI 1.033–1.558, *p* = 0.049) and *genus Eisenbergiella* (IVM: OR = 1.232, 95% CI 1.075–1.412, *p* = 0.003; WM: OR = 1.23, 95% CI 1.024–1.478, *p* = 0.027; MR-PRESSO: OR = 1.232, 95% CI 1.09–1.393, *p* = 0.009) gave rise to the development of IPF. In addition, there was a possible relationship between the 8 taxa and IPF. There was no evidence of pleiotropy or heterogeneity in the associations between these taxa and IPF (Figure 4; Supplementary Figure S5), and a leave-one-out analysis provided additional support for the consistency of these associations (Supplementary Figure S6). Ultimately, our analysis identified 2 probably pernicious taxa and 8 possible (1 pernicious and 7 defensive features) taxa that are linked with IPF.

**Table 4:** MR results of significant relationship between gut microbiota and IPF.

Exposures on IPF	SNPs	IVW		WM		MR-Presso		MR-Egger	
		OR (95% CI)	*p*	OR (95% CI)	*p*	OR (95% CI)	*p*	OR (95% CI)	*p*
C_Coriobacteriia	13	0.763 (0.602–0.968)	**0.026**	0.75 (0.554–1.016)	0.063	0.763 (0.602–0.968)	**0.046**	0.396 (0.156–1.006)	0.078
F_Acidaminococcaceae	5	0.735 (0.552–0.979)	**0.035**	0.771 (0.547–1.087)	0.138	0.735 (0.552–0.979)	0.103	0.531 (0.21–1.346)	0.275
F_Coriobacteriaceae	13	0.763 (0.602–0.968)	**0.026**	0.75 (0.547–1.028)	0.074	0.763 (0.602–0.968)	**0.046**	0.396 (0.156–1.006)	0.078
F_familyXIII	10	0.782 (0.613–0.998)	**0.048**	0.784 (0.577–1.065)	0.120	0.782 (0.665–0.92)	**0.016**	0.825 (0.359–1.894)	0.662
G_Ruminococcusgnavus	7	0.792 (0.629–0.996)	**0.046**	0.864 (0.678–1.1)	0.235	0.792 (0.629–0.996)	0.093	0.637 (0.226–1.79)	0.431
G_Blautia	10	1.269 (1.029–1.565)	**0.026**	1.362 (1.017–1.825)	**0.038**	1.269 (1.033–1.558)	**0.049**	1.181 (0.763–1.827)	0.476
G_Eisenbergiella	10	1.232 (1.075–1.412)	**0.003**	1.23 (1.024–1.478)	**0.027**	1.232 (1.09–1.393)	**0.009**	0.983 (0.36–2.682)	0.974
G_Holdemania	14	1.271 (1.095–1.476)	**0.002**	1.208 (0.985–1.481)	0.069	1.271 (1.095–1.476)	**0.008**	1.127 (0.724–1.756)	0.606
G_Hungatella	3	0.778 (0.629–0.962)	**0.021**	0.801 (0.609–1.055)	0.114	NA	NA	1.377 (0.399–4.747)	0.702
O_Coriobacteriales	13	0.763 (0.602–0.968)	**0.026**	0.75 (0.547–1.029)	0.074	0.763 (0.602–0.968)	**0.046**	0.396 (0.156–1.006)	0.078

All data with *p* < 0.05 are in bold. IPF, idiopathic pulmonary fibrosis; MR, Mendelian randomization; SNP, single nucleotide polymorphism; CI, confidence interval; IVW, inverse-variance-weighted; WM, weighted median; MR-PRESSO, MR pleiotropy residual sum and outlier; OR, odd ratio; P_: phylum; C_: class; O_: order; F_: family; G_: genus.

### 4.5. Causal relationship between gut microbiota and sarcoidosis

**Figure 6:**
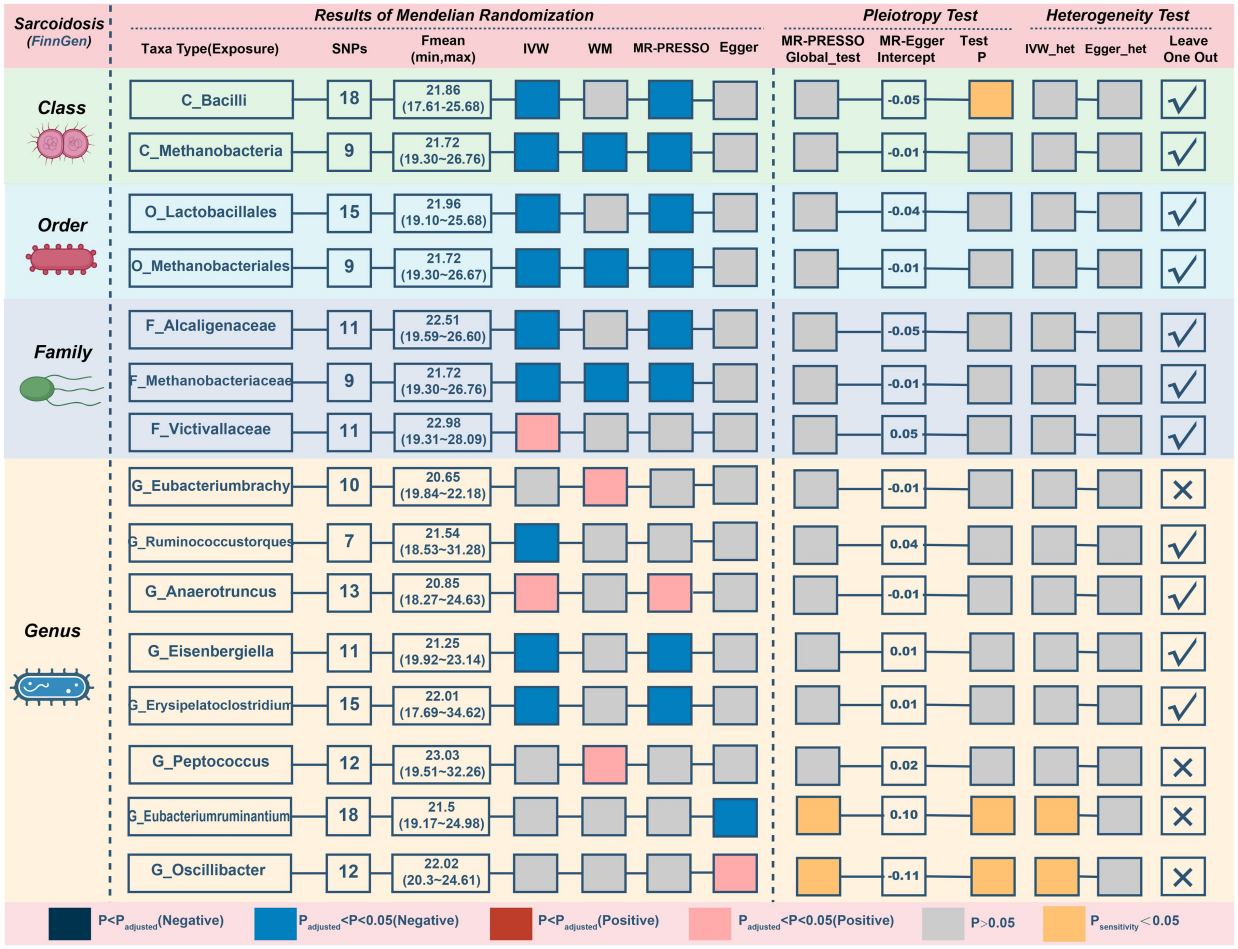
MR results and sensitivity analysis of significant relationship between gut microbiota and sarcoidosis. MR, Mendelian randomization; SNP, single nucleotide polymorphism; IVW, inverse-variance-weighted; MR-PRESSO, MR pleiotropy residual sum and outlier.

Next, we discover that three genetically predicted taxa (Figure 6; Table 5) *class Methanobacteria* (IVM: OR = 0.818, 95% CI 0.705–0.948, *p* = 0.008; WM: OR = 0.805, 95% CI 0.669–0.97, *p* = 0.022; MR-PRESSO: OR = 0.818, 95% CI 0.728–0.918, *p* = 0.009), *order Methanobacteriales* (IVM: OR = 0.818, 95% CI 0.705–0.948, *p* = 0.008; WM: OR = 0.805, 95% CI 0.664–0.978, *p* = 0.029; MR-PRESSO: OR = 0.818, 95% CI 0.728–0.918, *p* = 0.009) and *family Methanobacteriaceae* (IVM: OR = 0.818, 95% CI 0.705–0.948, *p* = 0.008; WM: OR = 0.805, 95% CI 0.667–0.973, *p* = 0.025; MR-PRESSO: OR = 0.818, 95% CI 0.728–0.918, *p* = 0.009) were significantly associated with sarcoidosis, and all three belonged to the same group. Furthermore, 12 taxa were discovered to have a possible association with sarcoidosis.

**Table 5:** MR results of significant relationship between gut microbiota and sarcoidosis.

Exposures on sarcoidosis	SNPs	IVW		WM		MR-Presso		MR-Egger	
		OR (95% CI)	*p*	OR (95% CI)	*p*	OR (95% CI)	*p*	OR (95% CI)	*p*
C_Bacilli	18	0.77 (0.625–0.947)	**0.013**	0.898 (0.67–1.201)	0.467	0.77 (0.638–0.929)	**0.014**	1.439 (0.814–2.545)	0.229
C_Methanobacteria	9	0.818 (0.705–0.948)	**0.008**	0.805 (0.669–0.97)	**0.022**	0.818 (0.728–0.918)	**0.009**	0.895 (0.499–1.604)	0.720
F_Alcaligenaceae	11	0.672 (0.508–0.89)	**0.005**	0.815 (0.546–1.217)	0.317	0.672 (0.512–0.884)	**0.017**	1.43 (0.396–5.168)	0.599
F_Methanobacteriaceae	9	0.818 (0.705–0.948)	**0.008**	0.805 (0.667–0.973)	**0.025**	0.818 (0.728–0.918)	**0.009**	0.895 (0.499–1.604)	0.720
F_Victivallaceae	11	1.177 (1.008–1.374)	**0.039**	1.165 (0.97–1.399)	0.101	1.177 (1.008–1.374)	0.066	0.838 (0.408–1.723)	0.642
G_Eubacteriumbrachy	10	1.095 (0.923–1.3)	0.298	1.267 (1.038–1.546)	**0.020**	1.095 (0.923–1.3)	0.325	1.221 (0.59–2.527)	0.606
G_Eubacteriumruminantium	18	1.154 (0.934–1.425)	0.185	1.173 (0.933–1.473)	0.171	1.154 (0.934–1.425)	0.202	0.444 (0.258–0.761)	**0.009**
G_Ruminococcustorques	7	0.603 (0.379–0.957)	**0.032**	0.734 (0.415–1.299)	0.289	0.603 (0.379–0.957)	0.076	0.328 (0.079–1.371)	0.187
G_Anaerotruncus	13	1.308 (1.019–1.679)	**0.035**	1.288 (0.917–1.808)	0.144	1.308 (1.069–1.601)	**0.023**	1.543 (0.743–3.203)	0.269
G_Eisenbergiella	11	0.846 (0.721–0.992)	**0.039**	0.828 (0.669–1.024)	0.082	0.846 (0.747–0.957)	**0.024**	0.775 (0.238–2.526)	0.682
G_Erysipelatoclostridium	15	0.801 (0.671–0.955)	**0.014**	0.802 (0.632–1.016)	0.068	0.801 (0.686–0.934)	**0.013**	0.733 (0.366–1.466)	0.396
G_Oscillibacter	12	0.964 (0.731–1.272)	0.797	1.024 (0.779–1.346)	0.864	0.964 (0.731–1.272)	0.802	2.98 (1.269–7)	**0.031**
G_Peptococcus	12	1.126 (0.973–1.303)	0.112	1.238 (1.02–1.504)	**0.031**	1.126 (0.978–1.296)	0.128	0.98 (0.558–1.719)	0.944
O_Lactobacillales	15	0.791 (0.629–0.995)	**0.046**	0.895 (0.65–1.233)	0.498	0.791 (0.642–0.975)	**0.046**	1.337 (0.739–2.42)	0.355
O_Methanobacteriales	9	0.818 (0.705–0.948)	**0.008**	0.805 (0.664–0.978)	**0.029**	0.818 (0.728–0.918)	**0.009**	0.895 (0.499–1.604)	0.720

All data with *p* < 0.05 are in bold. MR, Mendelian randomization; SNP, single nucleotide polymorphism; CI, confidence interval; IVW, inverse-variance-weighted; WM, weighted median; MR-PRESSO, MR pleiotropy residual sum and outlier; OR, odd ratio; P_: phylum; C_: class; O_: order; F_: family; G_: genus.

Three possible taxa exhibited significant pleiotropy or heterogeneity (Figure 6; Supplementary Figure S7) within the correlations (*class Bacilli, genus Eubacteriumruminium, and genus Oscillibacter*). The relationships of 2 possible taxa (*genus Eubacteriumbrachy and genus Peptococcus*) were also omitted since the leave-one-out analysis presented inconsistent findings (Supplementary Figure S8). After eliminating unstable traits, our analysis identified 3 probably defensive taxa and 7 possible taxa for sarcoidosis (2 pernicious and 5 defensive features).

### 4.6. Causal relationship between gut microbiota and pneumoconiosis

**Figure 7:**
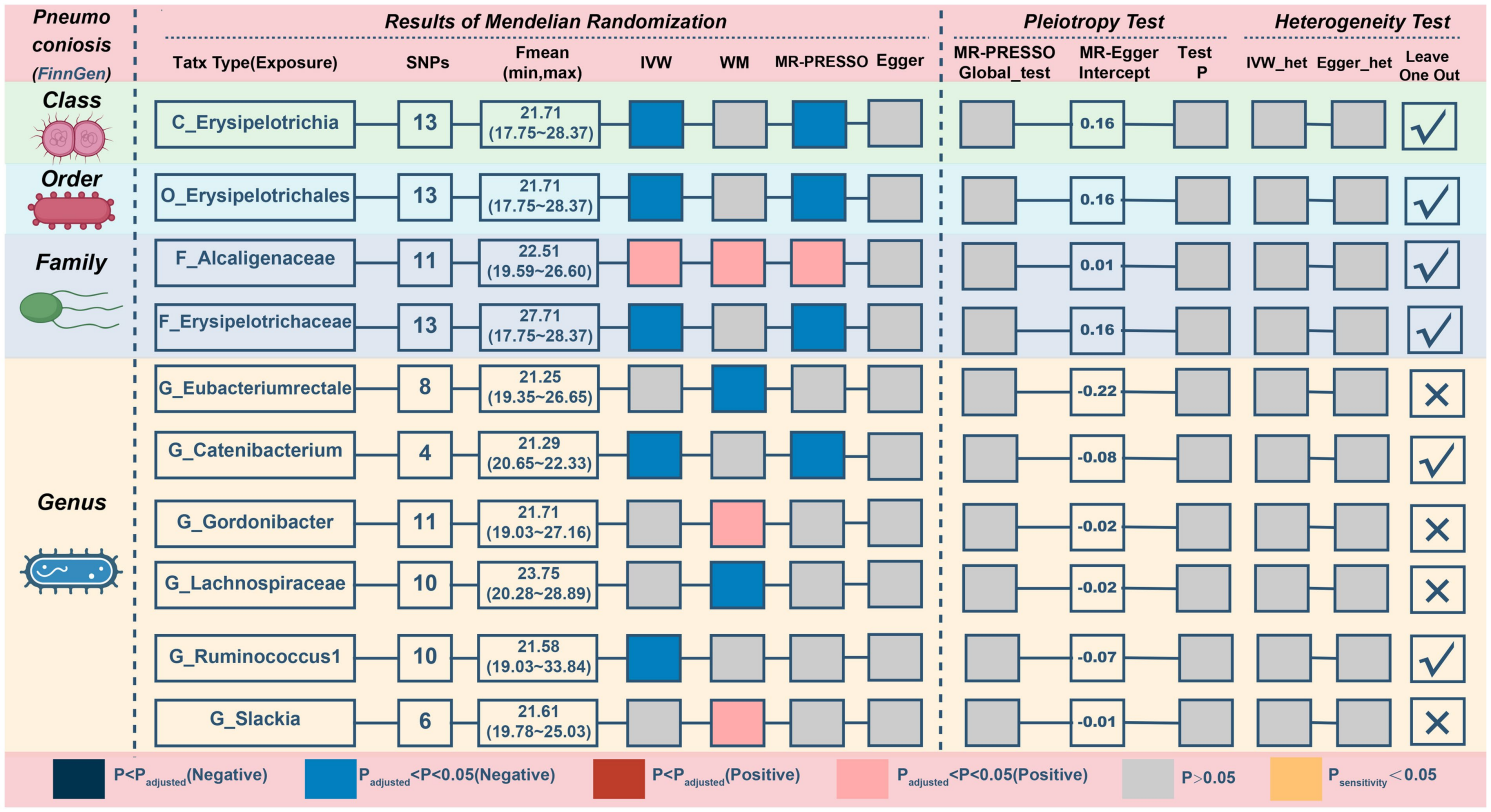
MR results and sensitivity analysis of significant relationship between gut microbiota and pneumoconiosis. MR, Mendelian randomization; SNP, single nucleotide polymorphism; IVW, inverse-variance-weighted; MR-PRESSO, MR pleiotropy residual sum and outlier.

In reference to the impact of gut microbiota on pneumoconiosis (Figure 7; Table 6), increasing levels of the *family Alcaligenaceae* contributed to disease formation (IVM: OR = 2.394, 95%CI 1.17–4.896, *p* = 0.017; WM: OR = 2.909, 95%CI 1.124–7.531, *p* = 0.028; MR-PRESSO: OR = 2.394, 95%CI 1.3–4.408, *p* = 0.019). Furthermore, there may have been a possible links between the 9 taxa and pneumoconiosis. No obvious pleiotropy or heterogeneity in the associations was found between these taxa and pneumoconiosis (Figure 7; Supplementary Figure S9). The links of four possible taxa (*genera Eubacteriumrectale, Gordonibacte, Lachnospiraceae, and Slackia*) were excluded, however, because the leave-one-out analysis produced conflicting results (Supplementary Figure S10). In the end, our analysis revealed that pneumoconiosis is associated with 1 probably hazardous taxon and 5 possibly protective taxa.

**Table 6:** MR results of significant relationship between gut microbiota and pneumoconiosis.

Exposures on Pneumoconiosis	SNPs	IVW		WM		MR-Presso		MR-Egger	
		OR (95% CI)	*p*	OR (95% CI)	*p*	OR (95% CI)	*p*	OR (95% CI)	*p*
C_Erysipelotrichia	13	0.393 (0.172–0.901)	**0.027**	0.406 (0.143–1.153)	0.090	0.393 (0.172–0.901)	**0.048**	0.029 (0.001–0.806)	0.061
F_Alcaligenaceae	11	2.394 (1.17–4.896)	**0.017**	2.909 (1.124–7.531)	**0.028**	2.394 (1.3–4.408)	**0.019**	2.105 (0.079–56.139)	0.667
F_Erysipelotrichaceae	13	0.393 (0.172–0.901)	**0.027**	0.406 (0.149–1.101)	0.077	0.393 (0.172–0.901)	**0.048**	0.029 (0.001–0.806)	0.061
G_Eubacteriumrectale	8	0.414 (0.149–1.149)	0.090	0.279 (0.084–0.929)	**0.038**	0.414 (0.149–1.149)	0.134	11.209 (0.579–217.15)	0.161
G_Catenibacterium	4	0.518 (0.305–0.879)	**0.015**	0.57 (0.298–1.093)	0.091	0.518 (0.386–0.695)	**0.022**	0.979 (0.001–741.033)	0.995
G_Gordonibacter	11	1.224 (0.877–1.707)	0.235	1.545 (1.003–2.382)	**0.049**	1.224 (0.902–1.659)	0.223	1.403 (0.343–5.748)	0.649
G_Lachnospiraceae	10	0.793 (0.484–1.3)	0.358	0.518 (0.275–0.979)	**0.043**	0.793 (0.484–1.3)	0.382	7.685 (0.772–76.458)	0.120
G_Ruminococcus1	10	0.471 (0.228–0.973)	**0.042**	0.763 (0.294–1.976)	0.577	0.471 (0.228–0.973)	0.073	1.063 (0.151–7.488)	0.953
G_Slackia	6	1.717 (0.941–3.134)	0.078	2.184 (1.009–4.723)	**0.047**	1.717 (1.019–2.894)	0.098	1.825 (0.037–89.29)	0.777
O_Erysipelotrichales	13	0.393 (0.172–0.901)	**0.027**	0.406 (0.147–1.121)	0.082	0.393 (0.172–0.901)	**0.048**	0.029 (0.001–0.806)	0.061

All data with *p* < 0.05 are in bold. MR, Mendelian randomization; SNP, single nucleotide polymorphism; CI, confidence interval; IVW, inverse-variance-weighted; WM, weighted median; MR-PRESSO, MR pleiotropy residual sum and outlier; OR, odd ratio; P_: phylum; C_: class; O_: order; F_: family; G_: genus.

## 5. Discussion

To the best of our knowledge, this is the first time that the causal links between gut microbiota and CRDs have been investigated meticulously using publicly available genetic databases. In our study, GWAS data for 196 taxa were subjected to a comprehensive MR analysis to explore the potential role of gut microbiota in the onset of CRDs. Based on extensive genetic data from over 3,504,473 European participants, we identified several gut microbial taxa, including 14 probable microbial taxa (i.e., *Haemophilus, Ruminiclostridium*, and *Blautia*) and 33 possible microbial taxa, that play significant roles in the development of CRDs.

Studies on the gut-lung axis in respiratory disorders such as asthma, COPD, and pulmonary fibrosis suggests that the variation of gut microbiota may potentially prevent or ameliorate these conditions. The plausible mechanisms encompass the modulation of chronic inflammation, the generation of short-chain fatty acids (SCFAs), and the regulation of extraintestinal T cell populations (Chunxi et al., 2020). For instance, the perturbed gut microbiota triggered by antibiotic use in individuals with asthma can be characterized as an exacerbated Th2, Th1/Th17 immune response and diminished Treg population (Russell et al., 2015). It has been reported that individuals with COPD exhibit decreased levels of histone deacetylase (HDACs), which could contribute to the amplification in inflammatory process. And the levels of HDACs could be governed by the gut-microbiota metabolites, specifically short-chain fatty acids (SCFAs; Qu et al., 2022).

For this study, a growing review of the literature revealed a potential association between the gut microbiota involved in this research and COPD. The proportions of *Bacteroides* and *Lachnoclostridium* were reported to increase in COPD and were even higher in acute exacerbation of COPD (Wu et al., 2021). Fine particulate matter (PM2.5) is acknowledged as the most important ambient air pollutant and has been associated with increased mortality and morbidity in COPD. The abundance of *Bacteroides* was found to increase in the high PM2.5 exposure group and comprises the greatest proportion of the gut microbiota in the COPD (Lin et al., 2022). These findings provide support for our study’s findings that Bacteroides and Lachnoclostridium may promote COPD development. *Bacteroidaceae* and *Bacteroides* are members of the same category and may aid in the formation of COPD across a similar mechanism. Our research also found that *Haemophilus* could render the development of COPD. These results are in line with recent studies that *Haemophilus* in the airways of COPD could prolong stable duration by increasing sputum IL-1 and TNF (tumor necrosis factor) (Wang et al., 2021) and that a decline in *Haemophilus* is linked to increased risk of mortality (Dicker et al., 2021). In addition, the preventative role of *Bacillales* collaborates with recent findings that the relative abundance of *Bacillales* was found to be lower in the high PM2.5 exposure group (Lin et al., 2022).

In terms of the effects of the three highly probable microbiota on asthma, the current study reveals that *Gammaproteobacteria* and *NB1-n* may promote development, whereas *Ruminiclostridium* has the opposite effect. These results are in line with a previous study showing that the abundance of *Gammaproteobacteria* was greater in urban schools with a greater asthma prevalence than in rural schools (Fu et al., 2021). *Tenericutes*, primarily ‘*NB1-n*’ (SILVA taxonomy) or ‘*RF3*’ (Greengenes taxonomy), indicated a decreased abundance in Pglyrp1−/− mice with a lower asthmatic response (Skennerton et al., 2016; Banskar et al., 2019). For *Ruminiclostridium,* a recent study found that intranasal delivery of rural dusts decreased eosinophils and plasma IgE levels in mice and contributed to a recovery of gut microbiota diversity and *Ruminiclostridium* in a mouse model, suggesting that exposure to *Ruminiclostridium* may promote allergy management (Yang et al., 2022).

Apart from CRD-related mortality from COPD (3.6% global prevalence) and asthma (3.0% global prevalence), interstitial lung disease and pulmonary sarcoidosis have been the second largest cause of death in high-income nations such as Europe and central Asia (Collaborators GBDCRD, 2020). Using MR analysis, we found that *Blautia* and *Eisenbergiella* have a protective effect against IPF. There has been little research on *Blautia* and IPF, despite studies showing an increase in lung cancer and lung tuberculosis. A possible explanation for this may be pulmonary structural changes in all these diseases (Liu et al., 2019; Naidoo et al., 2021). Recent research indicates that the abundance of *Eisenbergiella* is enhanced in a variety of connective tissue illnesses, including scleroderma and rheumatoid arthritis (Consortium I, 2022). It is widely known that connective tissue diseases are major causes of interstitial lung disease (ILD). Hence, we postulate that *Eisenbergiella* play a similar role in the pathogenesis of IPF as they do in CTD but this hypothesis remains to be validated.

For sarcoidosis, three taxa with causal links all belong to the sort of *Methanobacteria*. *Methanobacteria* groups are commonly found in anaerobic environments, such as soils and the digestive tracts of animals, which comprise critical elements of methanogenic archaea and are linked to the development of diseases, including cancer (Cai et al., 2022). Considering the significance of the three MR analyzes, it is essential to perform further studies to determine the specific involvement of *Methanobacteria* in sarcoidosis. To evaluate the impact of gut microbiota on pneumoconiosis, we selected patients exposed to asbestos and other mineral fibers due to their prevalence and larger number of cases. In this study, the *Alcaligenaceae family* was declared to be hazardous, and this finding was similar to that of Diana C’s study, which indicated that *Alcaligenaceae* emerged solely in the Tanner group compared to the control group and were deemed pathogenic bacteria. (Castellanos-Arévalo et al., 2015). Markedly, to obtain a stringent and trustworthy conclusion, we discarded the significant taxa with considerable pleiotropy or heterogeneity that could have influenced the strength of the causal links (Hemani et al., 2018; Verbanck et al., 2018).

It is equally important to recognize the limitations of our study. First, the majority of patients in the GWAS summary data utilized in our study were of European heritage and only a tiny fraction of the gut microbiota data were gathered from other ethnic groups. This may result in biased estimates and we must exert caution when extrapolating our findings to other ethnicities. Second, expanding the sample size is essential for achieving a more precise estimation of the link between gut microbiota and CRDs as there is the potential for estimation bias resulting from the relatively small sample size of gut microbiota. Third, due to a lack of individual data, bacterial taxa were only evaluated with summary statistics. To investigate potential differences between groups, additional population stratification analyzes (e.g., by gender, age) may be conducted. Considering the substantial influence of diet on gut microbiota and the variations in dietary patterns (Mediterranean, plant-based or high-fat) across populations, it is imperative to account for diet when validating these potential associations in future researches (Beam et al., 2021). Finally, since MR analysis is predicated on untestable hypotheses, further clinical validation studies are necessary to ascertain the therapeutic value of microbial species.

## 6. Conclusion

In conclusion, we systematically evaluated the potential relationship between the gut microbiota and five prevalent CRDs and discovered 14 probable relationships and 33 possible relationships for the first time. This study highlights the probable causative role of gut microbes in the genesis of CRDs, indicating to clinicians that modifying gut microbiota may be an option for disease prevention.

## Data availability statement

The original contributions presented in the study are included in the article/Supplementary material, further inquiries can be directed to the corresponding authors.

## Ethics statement

Ethical review and approval was not required for the study on human participants in accordance with the local legislation and institutional requirements. The patients/participants provided their written informed consent to participate in this study.

## Author contributions

HS conceived of the study, analyzed the data, and drafted the manuscript. TZ conducted the data analysis and authored the paper. RG and TZ gathered and sanitized the data. LS and HF conceived the study, oversaw its execution, and edited the manuscript. All authors contributed to the article and approved the submitted version.

## Funding

This study was funded by the China National Natural Science Foundation (no. 81971878).

## Conflict of interest

The authors declare that the research was conducted in the absence of any commercial or financial relationships that could be construed as a potential conflict of interest.

## Publisher’s note

All claims expressed in this article are solely those of the authors and do not necessarily represent those of their affiliated organizations, or those of the publisher, the editors and the reviewers. Any product that may be evaluated in this article, or claim that may be made by its manufacturer, is not guaranteed or endorsed by the publisher.

## Footnotes

^1^ http://www.mibiogen.org/

^2^ https://www.finngen.fi/

^3^ http://www.phenoscanner.medschl.cam.ac.uk/
