# Supplementary material for: The associations between gut microbiota and chronic respiratory diseases: a Mendelian randomization study
Source: Front Microbiol. 2023 Jun 2;14:1200937. doi: 10.3389/fmicb.2023.1200937 (PMC10272395; doi:10.3389/fmicb.2023.1200937)
Supplement: Supplementary file 2 [file Data_Sheet_1.PDF]

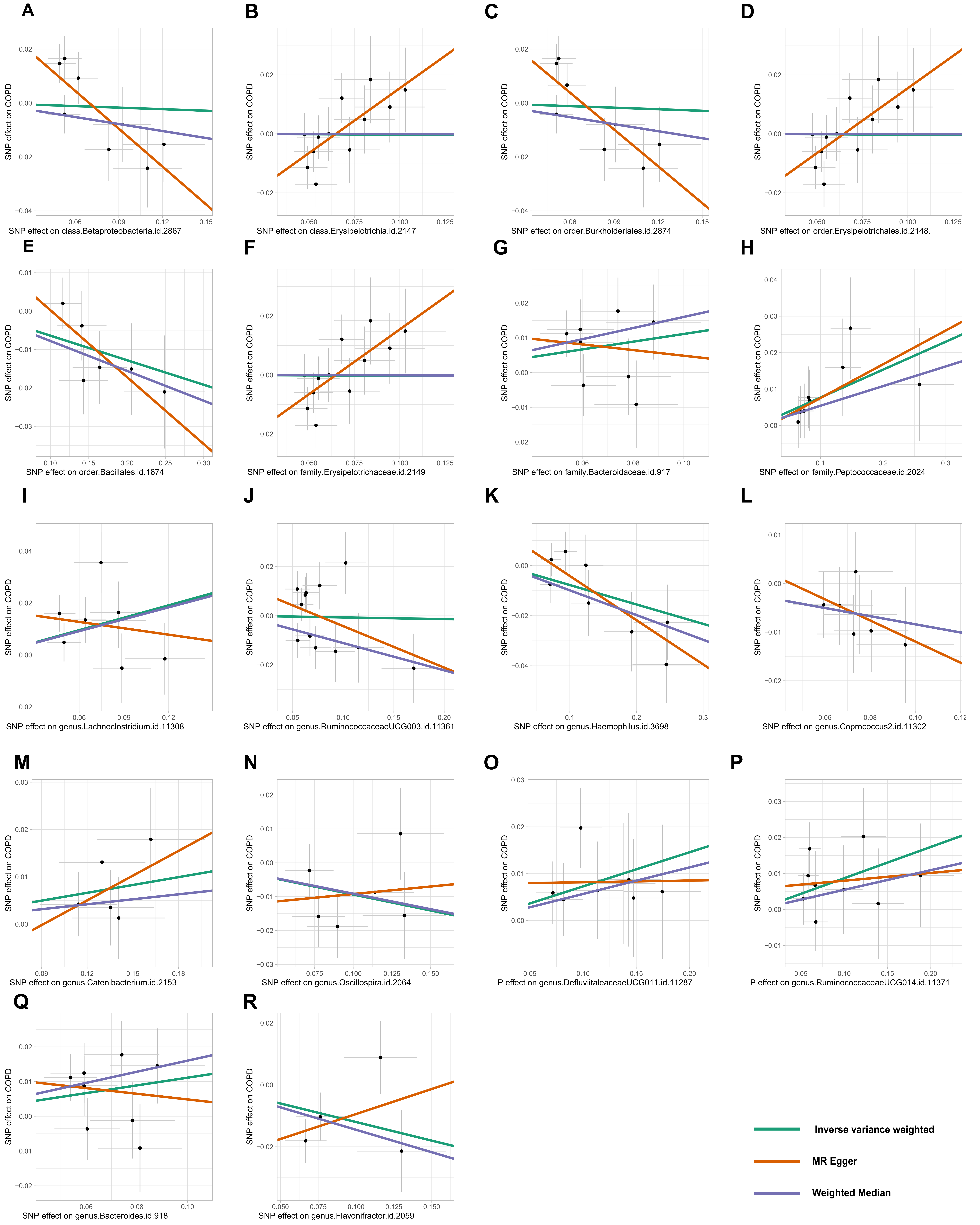

Figure S1 Scatterplot displaying 18 significant relationships of gut microbiota with COPD (chronic pulmonary obstructive disease). The slope of each line corresponds to the influence estimated by various models.

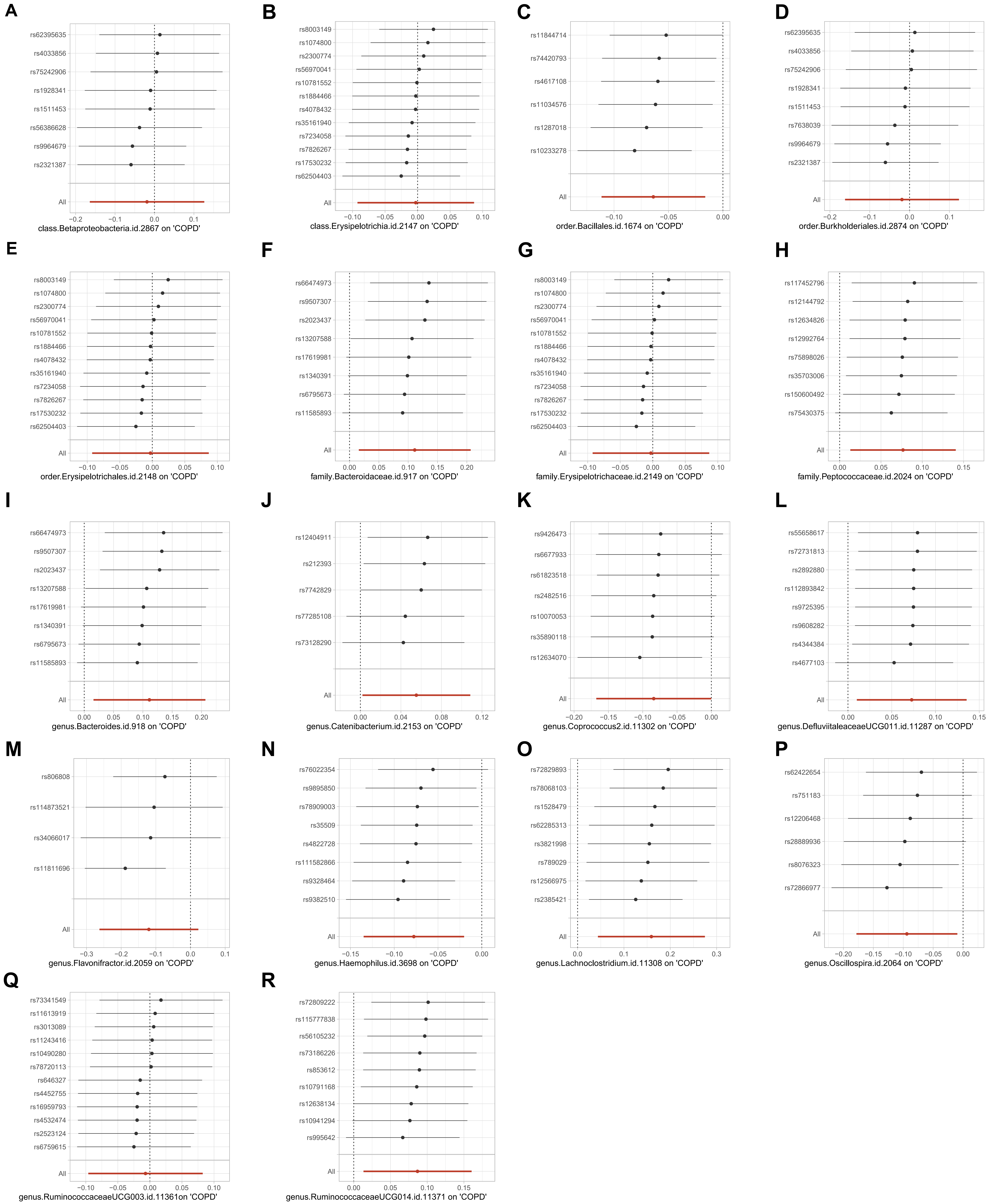

Figure S2 Leave-one-out plots of MR analysis results for 18 gut microbiotas on COPD (chronic obstructive pulmonary disease)

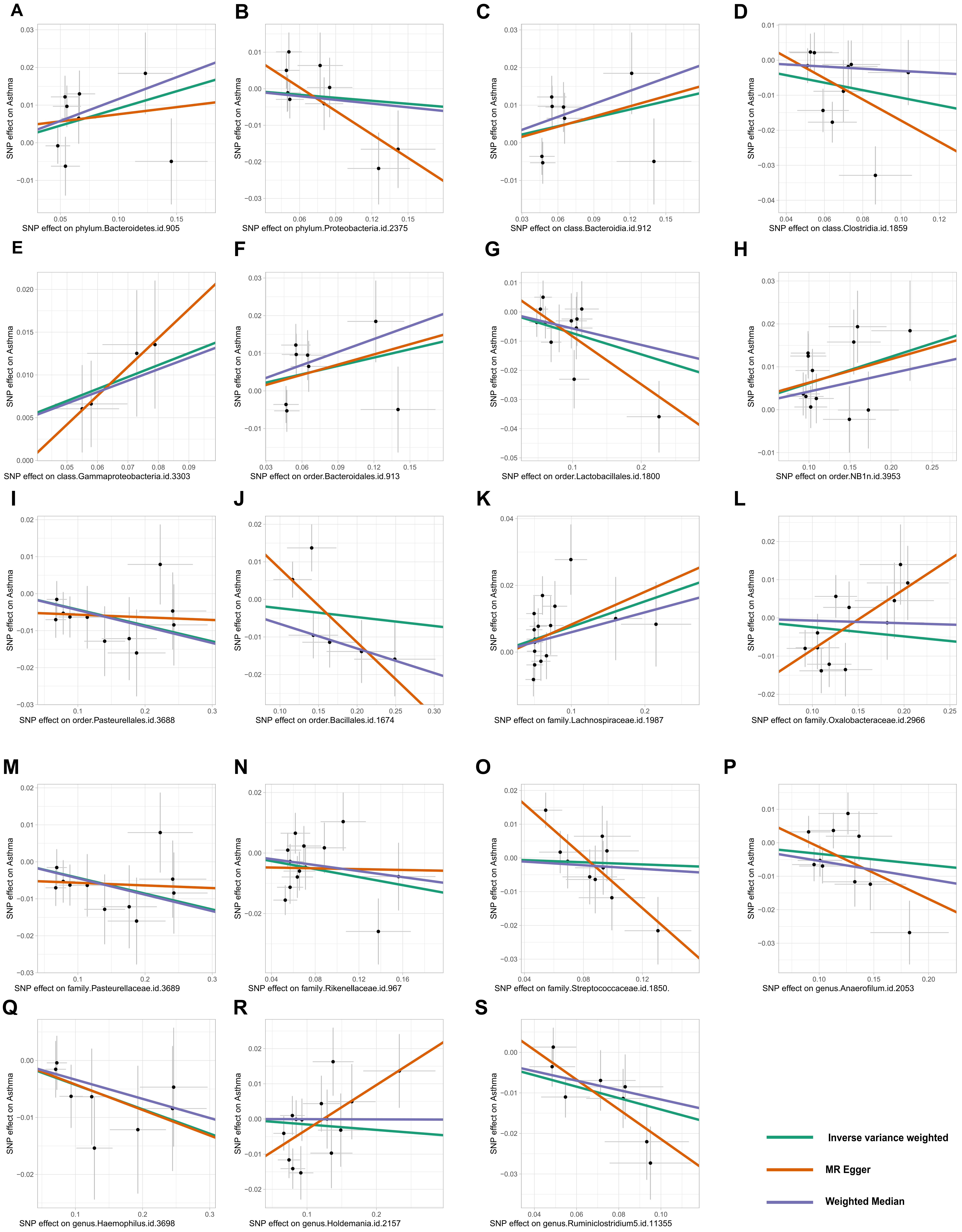

Figure S3 Scatterplot displaying 19 significant relationships of gut microbiota with asthma. The slope of each line corresponds to the influence estimated by various models.

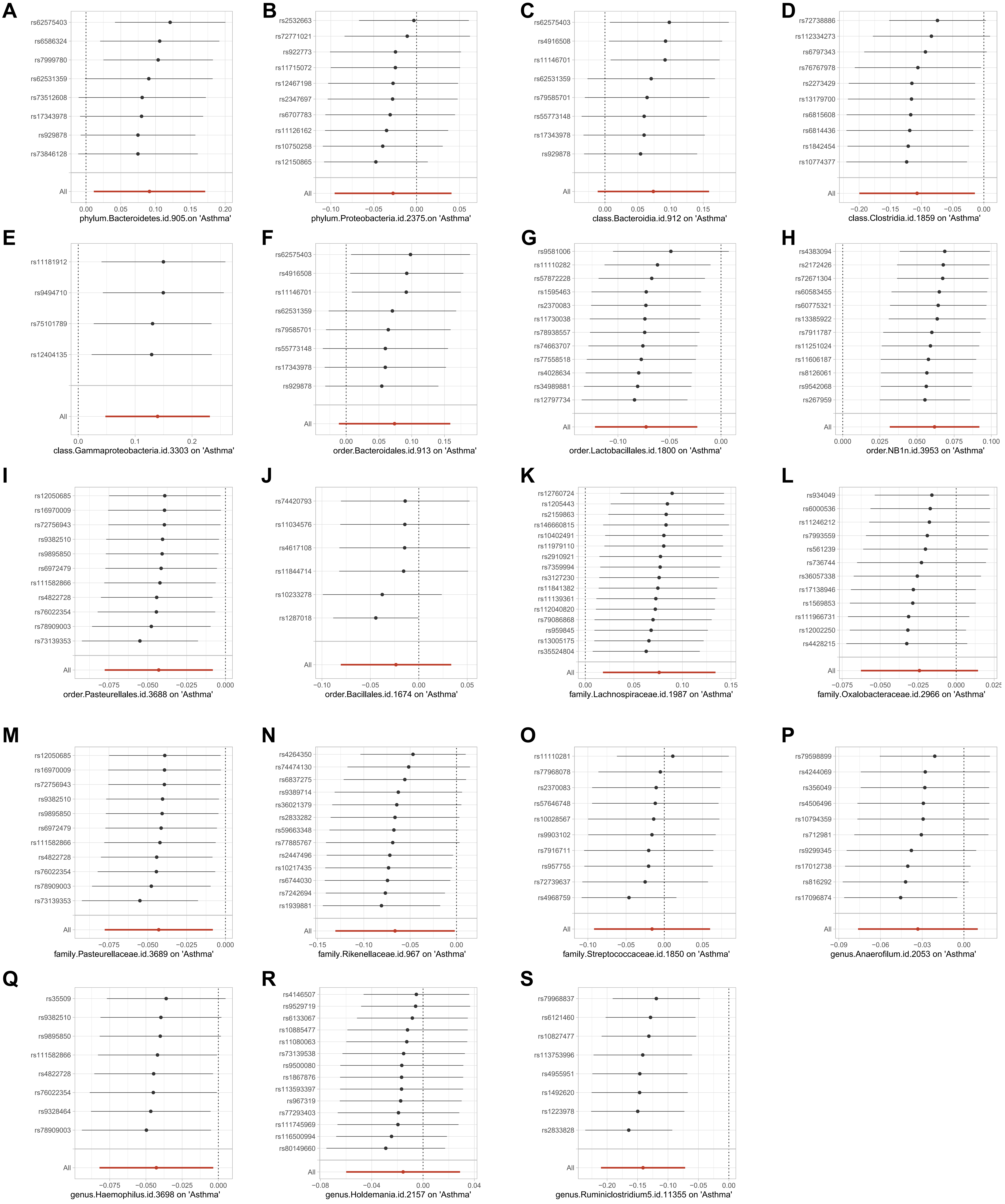

Figure S4 Leave-one-out plots of MR analysis results for 19 gut microbiotas on asthma.

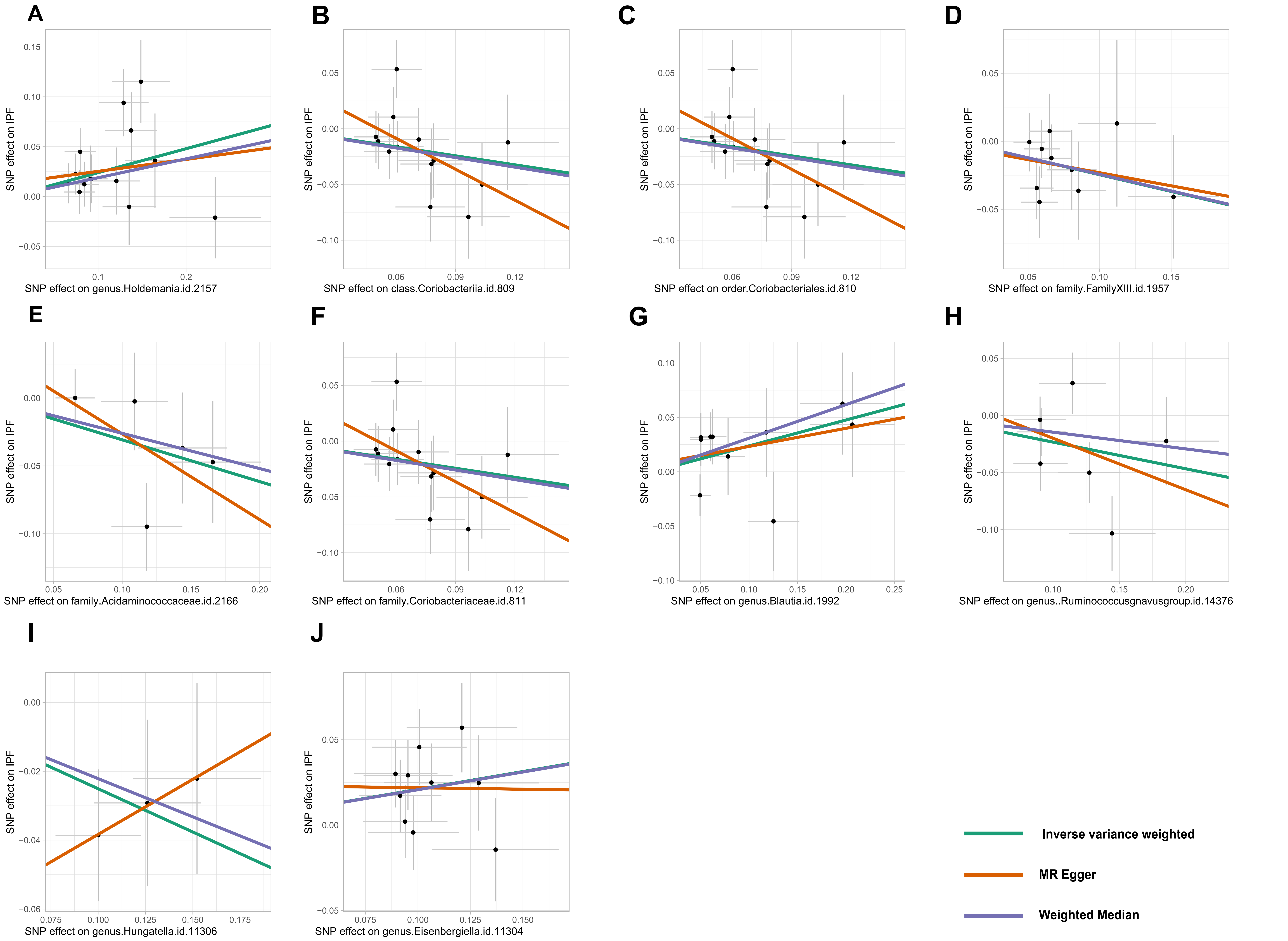

Figure S5 Scatterplot displaying 10 significant relationships of gut microbiota with IPF(idiopathic pulmonary fibrosis). The slope of each line corresponds to the influence estimated by various models.

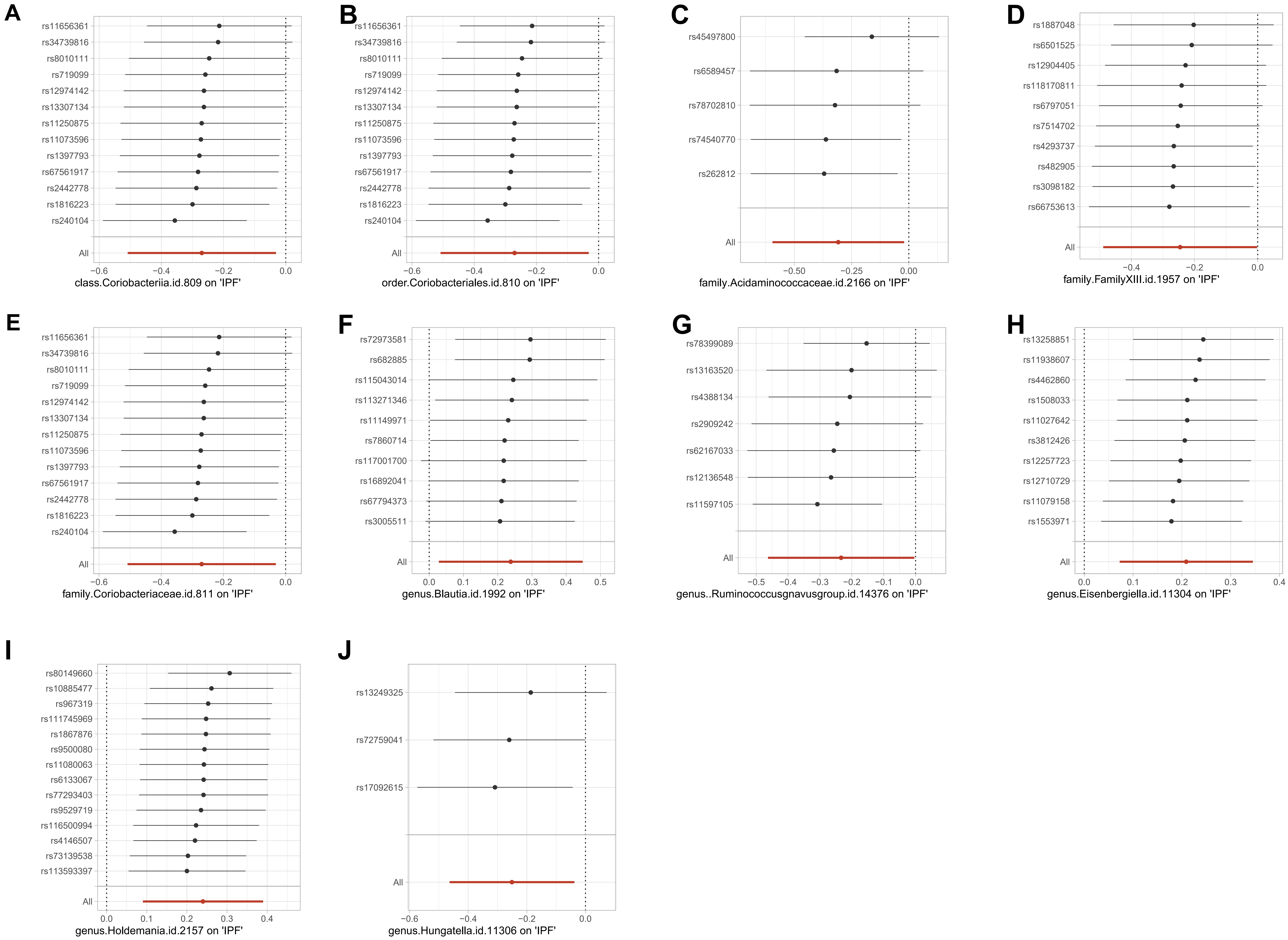

Figure S6 Leave-one-out plots of MR analysis results for 10 gut microbiotas on IPF (idiopathic pulmonary fibrosis)

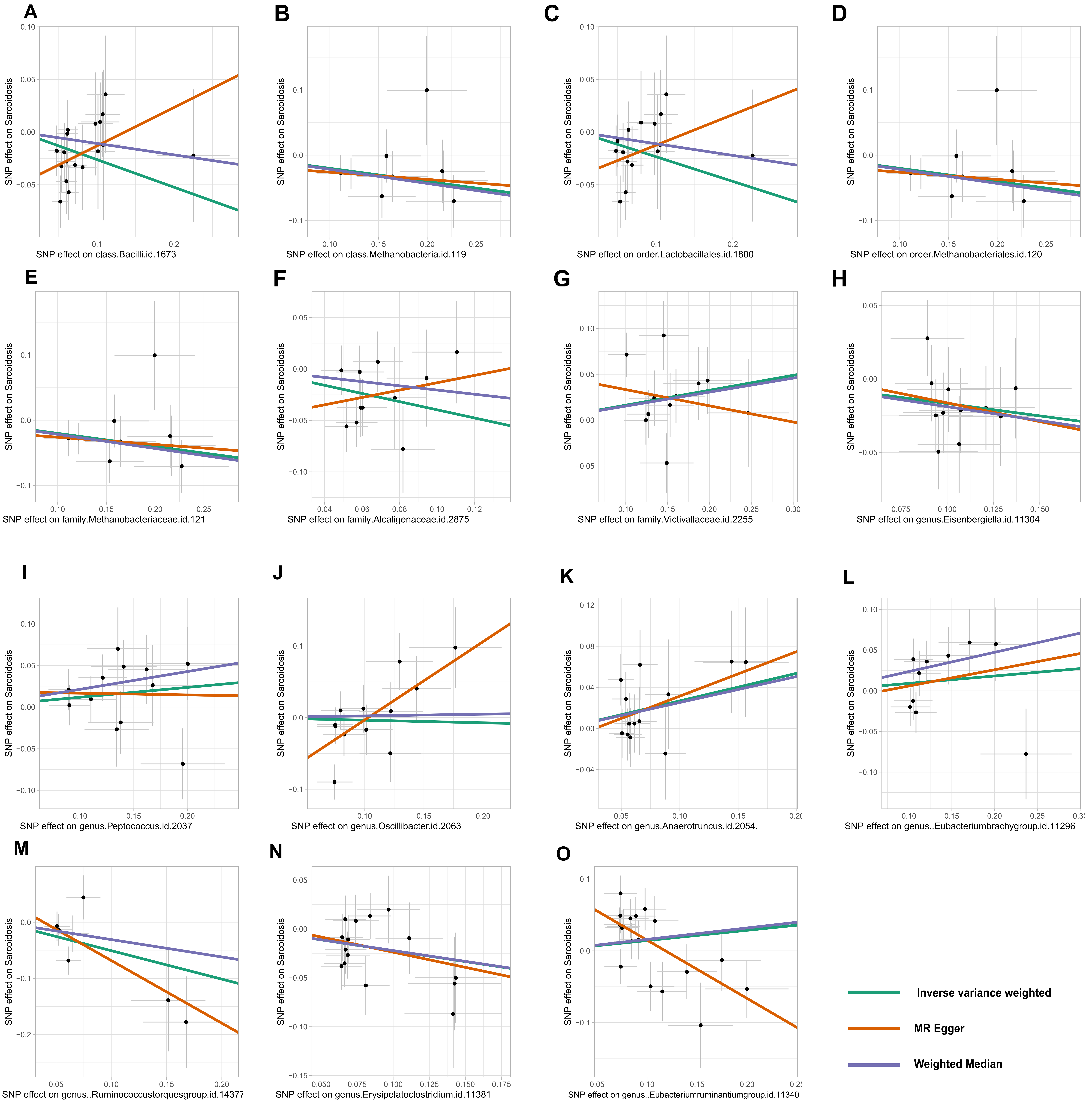

Figure S7 Scatterplot displaying 15 significant relationships of gut microbiota with Sarcoidosis. The slope of each line corresponds to the influence estimated by various models.

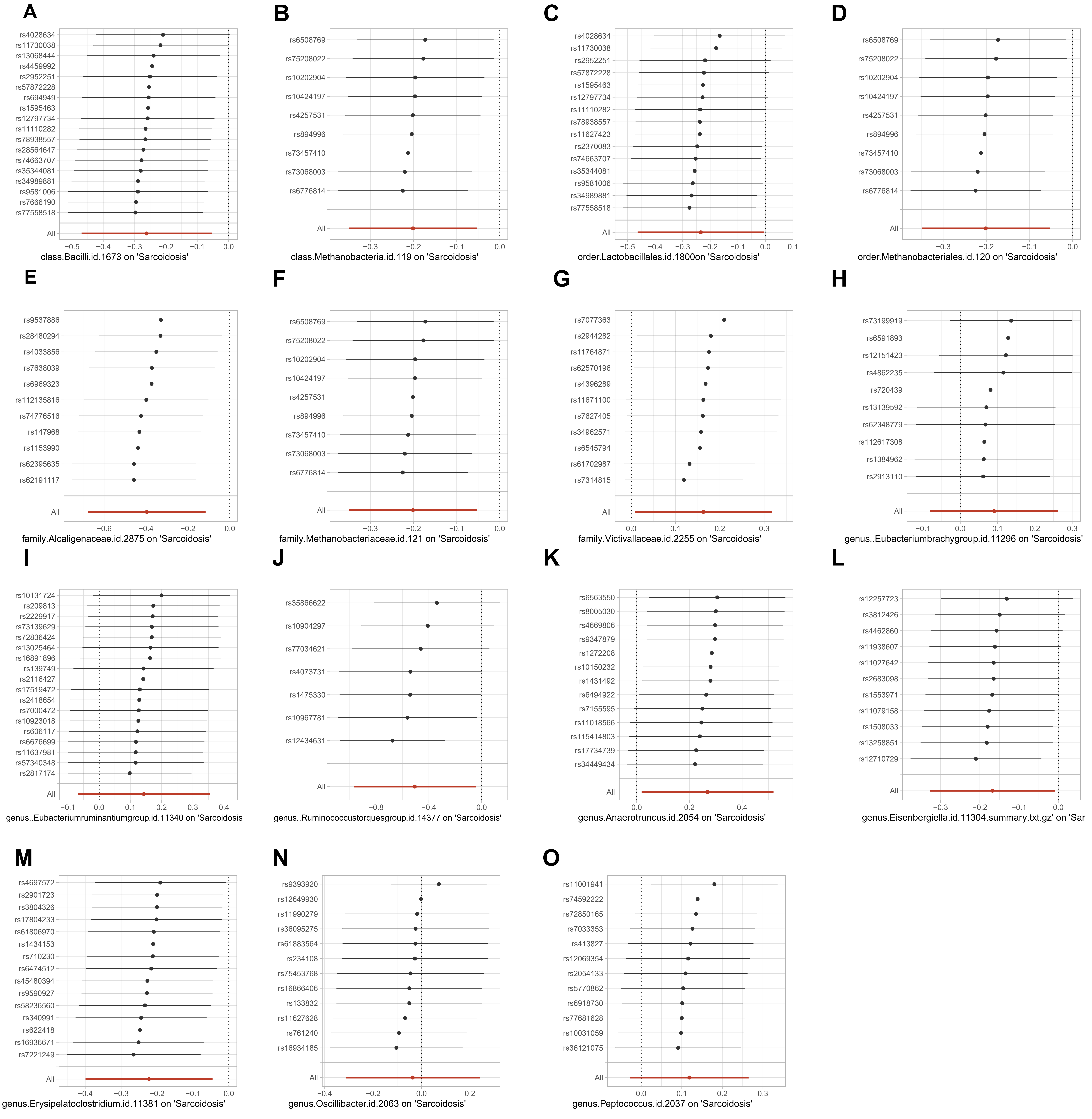

Figure S8 Leave-one-out plots of MR analysis results for 15 gut microbiotas on sarcoidosis.

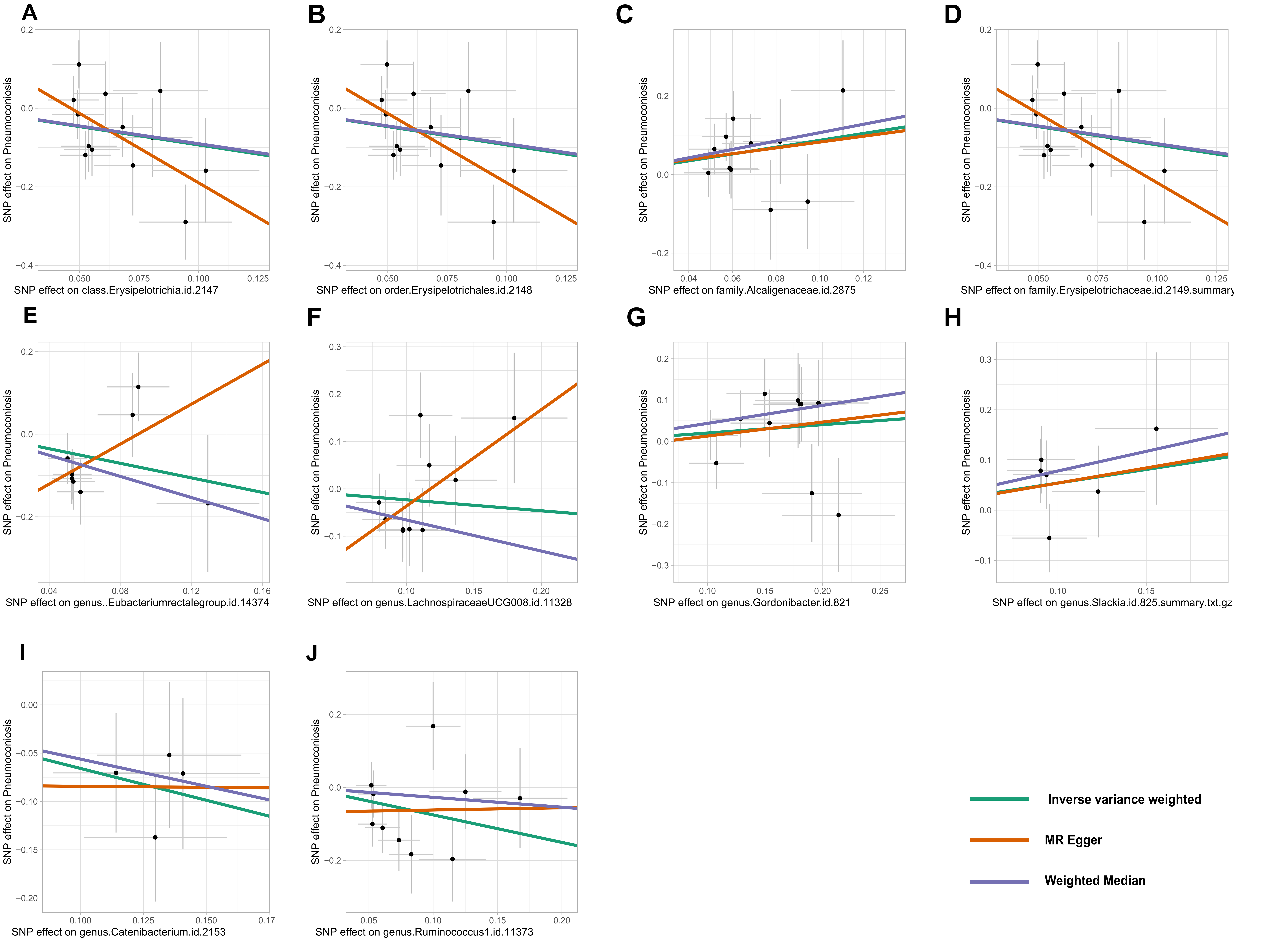

Figure S9 Scatterplot displaying 10 significant relationships of gut microbiota with pneumoconiosis. The slope of each line corresponds to the influence estimated by various models.

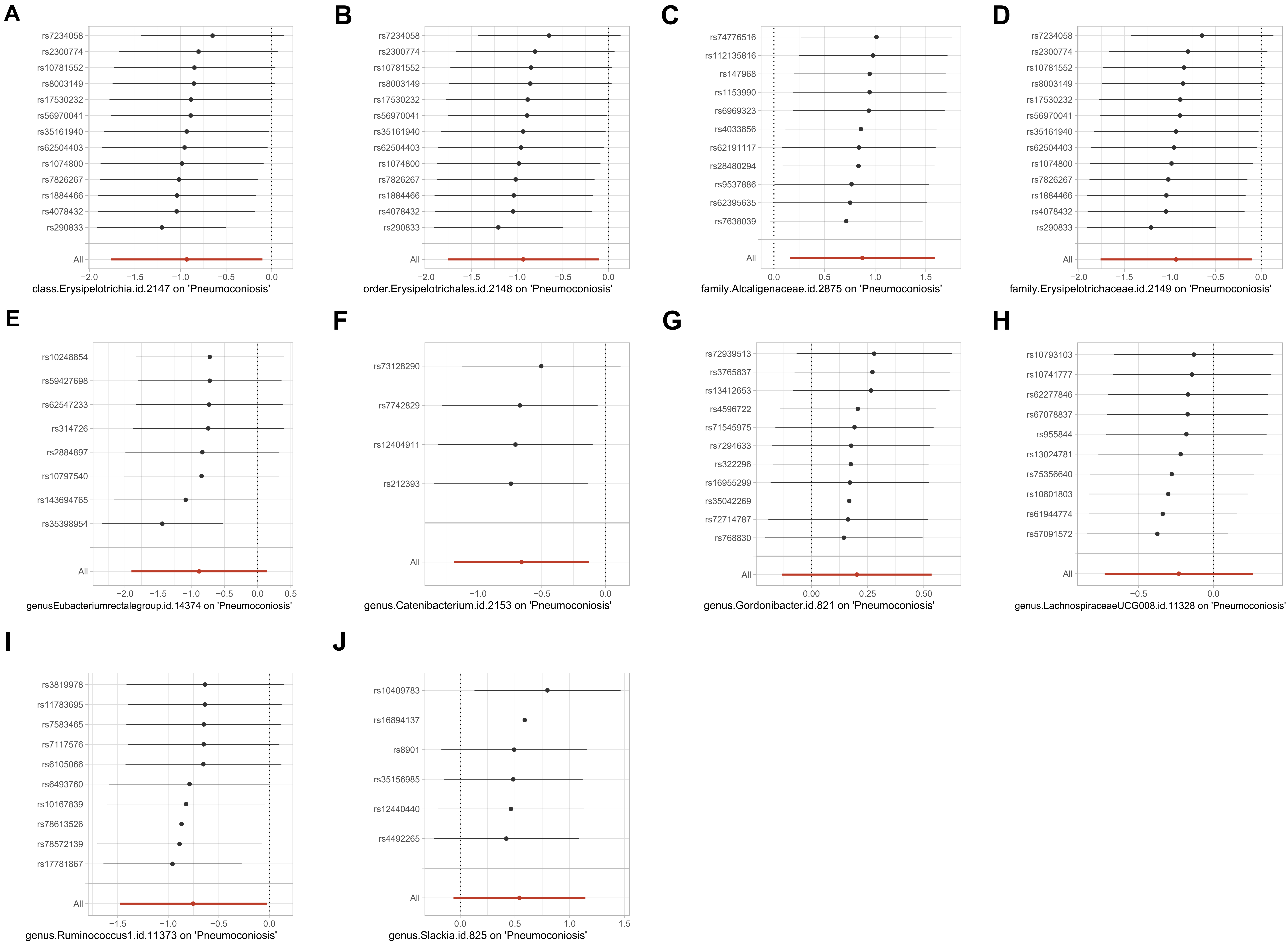

Figure S10 Leave-one-out plots of MR analysis results for 10 gut microbiotas on pneumoconiosis.
